# Azathioprine promotes intestinal epithelial cell differentiation into Paneth cells and alleviates ileal Crohn’s disease severity

**DOI:** 10.1038/s41598-024-63730-4

**Published:** 2024-06-05

**Authors:** Mohab Ragab, Heidi Schlichting, Maren Hicken, Patricia Mester, Misa Hirose, Larissa N. Almeida, Lea Christiansen, Saleh Ibrahim, Hauke Christian Tews, Senad Divanovic, Christian Sina, Stefanie Derer

**Affiliations:** 1 Institute of Nutritional Medicine, University Hospital Schleswig-Holstein, Campus Lübeck, Ratzeburger Allee 160, 23562 Lübeck, Germany; 2 Department of Internal Medicine I, Gastroenterology, Hepatology, Endocrinology, Rheumatology and Infectious Diseases, University Hospital, Regensburg, Germany; 3 Lübeck Institute of Experimental Dermatology and Center for Research On Inflammation of the Skin, University of Lübeck, Lübeck, Germany; 4 College of Medicine and Health Sciences, Khalifa University, Abu Dhabi, United Arab Emirates; 5 Department of Pediatrics, University of Cincinnati College of Medicine, Cincinnati, OH USA; 6 Division of Immunobiology, Cincinnati Children’s Hospital Medical Center, Cincinnati, OH USA; 7 Institute of Nutritional Medicine and 1st Department of Medicine, Division of Nutritional Medicine, University Hospital Schleswig-Holstein, Campus Lübeck, Lübeck, Germany

**Keywords:** Crohn's disease, Mechanisms of disease

## Abstract

Paneth cells (PCs), a subset of intestinal epithelial cells (IECs) found at the base of small intestinal crypts, play an essential role in maintaining intestinal homeostasis. Altered PCs function is associated with diverse intestinal pathologies, including ileal Crohn’s disease (CD). CD patients with ileal involvement have been previously demonstrated to display impairment in PCs and decreased levels of anti-microbial peptides. Although the immunosuppressive drug Azathioprine (AZA) is widely used in CD therapy, the impact of AZA on IEC differentiation remains largely elusive. In the present study, we hypothesized that the orally administered drug AZA also exerts its effect through modulation of the intestinal epithelium and specifically via modulation of PC function. AZA-treated CD patients exhibited an ileal upregulation of AMPs on both mRNA and protein levels compared to non-AZA treated patients. Upon in vitro AZA stimulation, intestinal epithelial cell line MODE-K exhibited heightened expression levels of PC marker in concert with diminished cell proliferation but boosted mitochondrial OXPHOS activity. Moreover, differentiation of IECs, including PCs differentiation, was boosted in AZA-treated murine small intestinal organoids and was associated with decreased D-glucose consumption and decreased growth rates. Of note, AZA treatment strongly decreased *Lgr5* mRNA expression as well as *Ki67* positive cells. Further, AZA restored dysregulated PCs associated with mitochondrial dysfunction. AZA-dependent inhibition of IEC proliferation is accompanied by boosted mitochondria function and IEC differentiation into PC.

## Introduction

The small intestine (SI) is lined by diverse subsets of epithelial cells that vary in function and metabolic requirements. Intestinal epithelium is a highly dynamic compartment that undergoes frequent renewal every 3–5 days^1^. Paneth cells (PCs) which reside at the base of intestinal crypts have a long lifespan (> 30 days) and play a multifaceted role in mucosal immunity and intestinal homeostasis^1,2^. For example, PCs are major source of antimicrobial proteins and peptides (AMPs) that are present in the lumen of the small intestine^3^. The acidophilic granules that distinguish PCs from other IECs contain various immune-modulatory proteins and peptides including α-defensins, lysozyme, secretory phospholipase A2 and angiogenin-4^4–7^. PC granules’ secretion is initiated in response to various stimuli including cholinergic agonists and microbial products. In addition, AMPs have the ability to shape intestinal microbiota^8–10^. Vaishnava et al*.* reported that MyD88-dependent toll-like receptor (TLR) activation is the mechanism by which PCs exert their effects on luminal bacteria^8^. In another study, mice expressing a human α-defensin 5 (DEFA5) had low Firmicutes/Bacteroidetes ratio, while mice deficient in matrix metalloproteinase 7 (*Mmp7*), a gene encoding defensins activating mediator, showed the opposite^9^. Another crucial function that has been attributed to PCs is providing the niche for the maintenance of intestinal stem cells (ISCs) through releasing epidermal growth factor (EGF), transforming growth factor alpha (TGF-α), and wingless-related integration site (Wnt3)^11^.

Altered PCs function is associated with inflammatory bowel disease (IBD), and specifically Crohn`s disease (CD). Patients with ileal CD involvement exhibit reduced PC numbers as well as diminished α-defensins levels^12,13^. Mitochondrial function is a significant contributor to the metabolic balance required for intestinal epithelial cell (IEC) differentiation and proliferation. Disturbances in mitochondrial function can lead to intestinal inflammatory disorders such as IBD^14–16^. Aberrant PC function is linked with inflammation-associated mitochondrial dysfunction^15^. IEC-specific deletion of the mitochondrial chaperone Hsp60 resulted in loss of IEC differentiation and the generation of dysfunctional PCs^15^. In addition, absence of prohibitin 1 (*Phb1*), a critical inner mitochondrial membrane protein, in intestinal epithelium led to the development of spontaneous ileitis in mice^17^. Furthermore, we have previously demonstrated the importance of p32, an essential mitochondrial oxidative phosphorylation (OXPHOS)-stabilizing protein, in goblet cell differentiation in patients with ulcerative colitis^16^.

Azathioprine (AZA) is used to treat IBD patients over the past five decades^18,19^. AZA belongs to the family of thiopurines which are known as de novo purine synthesis suppressors^20^. The impact of AZA on immune cells was extensively investigated since it first came out in 1956^21,23^. Through interfering with DNA synthesis, AZA decreases the numbers of highly proliferating T and B cells^24,25^. Since AZA possesses a glucocorticoid-sparing effect, one of its primary indication is the induction and maintenance of remission upon glucocorticoids withdrawal^26^. Nevertheless, the effect of AZA on the highly proliferating intestinal epithelium is poorly understood. Of note, we showed that UC patients treated with AZA exhibit an increase in colonic p32 expression, suggesting that AZA acts through overcoming mitochondrial dysfunction^16^. As a result, AZA paves the way to correct the imbalance between proliferation and differentiation in IECs and ultimately promotes the resolution of inflammation. Moreover, AZA treatment can restore epithelial barrier function by influencing genes involved in tight junction regulation^27^. Specifically, high permeability of T-84 cells, a human colonic carcinoma cell line, treated with TNF-α and IFN-γ was lessened upon incubation with AZA or its metabolite mercaptopurine (6-MP)^27^.

Based on the previous studies, we hypothesized that due to its potent anti-proliferative properties, AZA could potentially enhance the differentiation of IECs. Hence, in this study we investigated how AZA impacts small intestinal epithelium with focus on PCs in the context of CD.

## Results

### Azathioprine treatment rescues Paneth cells’ function in patients with Crohn’s disease (CD)

**Figure 1:**
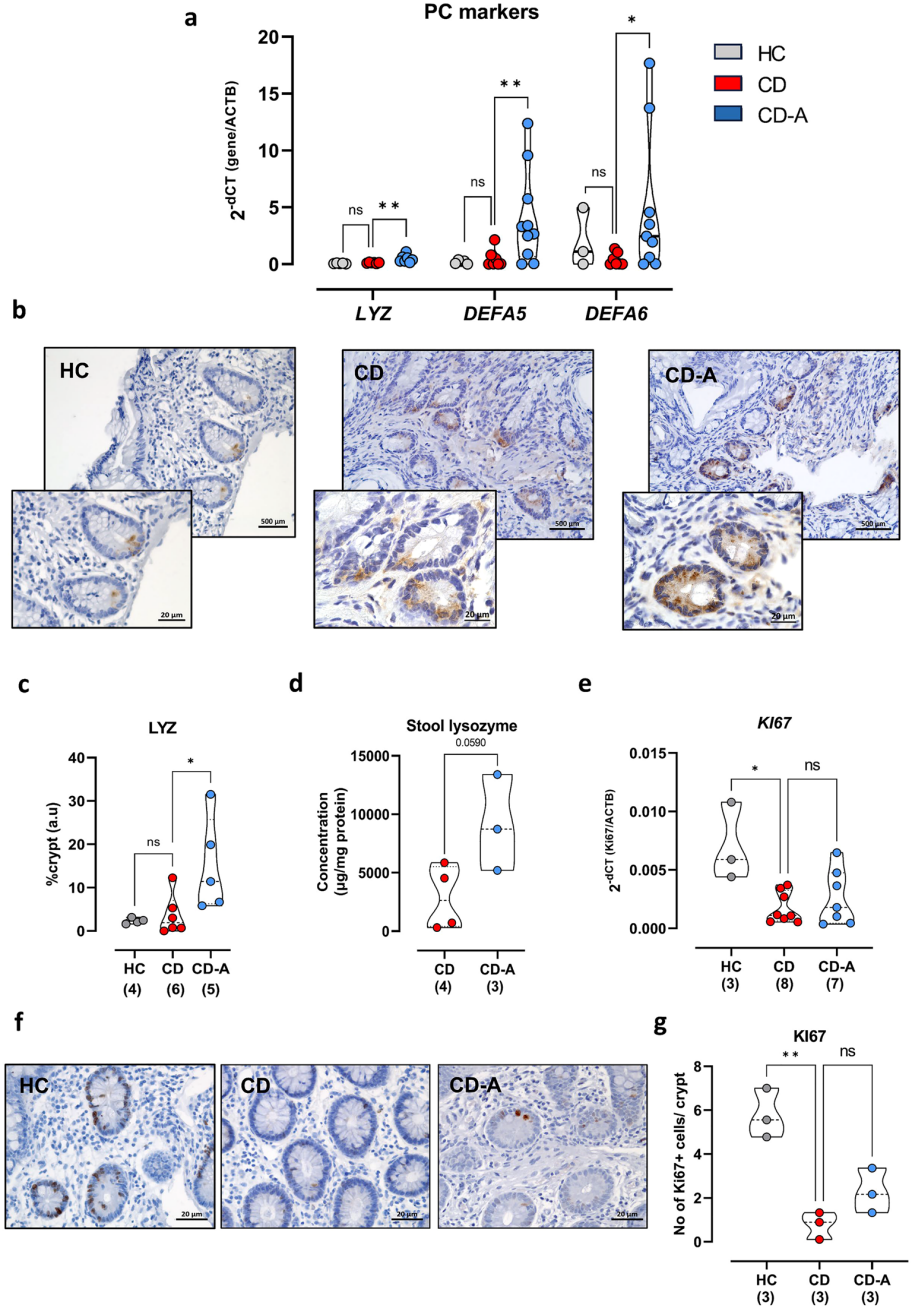
Levels of Paneth cells markers in CD patients and hospitalized controls. (**a**) mRNA expression of *LYZ*, *DEFA5* and *DEFA6* in ileal samples from CD patients and hospitalized controls. (**b**) Microscopic images of IHC staining of LYZ in ileal samples from CD patients and healthy controls. Scale bar = 500, 20 µm (20x, 63x). (**c**) Intensity of the staining per crypt was measured using ImageJ software. (**d**) Lysozyme level was measured in fecal samples from CD patients treated or not treated with AZA by ELISA. (**e**) qPCR was performed to measure mRNA levels of *KI67* in ileal biopsies CD patients and controls. (**f**) Representative images of ileal KI67 staining in CD patients and controls. Scale bar = 20 µm (63x). (**g**) Numbers of Ki67 + cells were counted using ImageJ software. (**a**,**c**,**e**,**g**) Kruskal–Wallis test; (**d**) Unpaired t test with Welch’s correction; results are shown as truncated violin plots with median represented as dashed line. *p < 0.05, **p < 0.01. HC: hospitalized controls; CD: non-AZA treated CD patients; CD-A: azathioprine-treated CD patients; *DEFA5*, 6: alpha-defensin 5, 6; *LYZ*: lysozyme.

AZA treatment is effective in active CD and also in maintaining remission^28,29^. As PCs dysfunction is hypothesized to trigger CD, we examined ex vivo the effect of AZA on PC function in CD patients and hospitalized healthy controls (Tables 1, 2, 3, 4). In qPCR experiments utilizing ileal biopsy samples from CD patients and controls, AZA-treated patients displayed a significant upregulation of *LYZ* expression compared to CD patients not treated with AZA (Fig. 1a). PCs secrete diverse anti-microbial peptides, mainly alpha-defensins such as DEFA5 and DEFA6^30^. Notably, ileal biopsies from AZA-treated patients had significantly increased mRNA expression of *DEFA5* and *DEFA6* (Fig. 1a). On the other hand, mRNA levels of PC markers did not differ between controls and CD patients not treated with AZA (Fig. 1a).

**Table 1:** Clinical characteristics of hospitalized controls included in the study.

	*LYZ/DEFA5* (qPCR)		*DEFA6* (qPCR), KI67 (qPCR,IHC)
Total number of patients	6		3
Male/female	2 (33.3)/4 (66.7)		1 (33.3)/2 (66.7)
Age (mean ± SD)	52.3 ± 16.2		58.3 ± 11

**Table 2:** Clinical characteristics of CD patients included for qPCR analysis.

	*LYZ*		*DEFA5/6*		*KI67/IL1β/TNF-α/IL8/IL6*	
Total number of patients	13		17		15	
AZA therapy	( −) 6 (46.2)	( +) 7 (53.8)	( −) 7 (41.2)	( +) 10 (58.8)	( −) 8 (53.3)	( +) 7 (46.7)
Disease activity						
Remission	3 (50)	6 (85.1)	3 (42.9)	7 (70)	3 (37.5)	4 (57.1)
Active	3 (50)	1 (14.3)	4 (57.1)	3 (30)	5 (62.5)	3 (42.9)
Male/female	2 (33.3)/4 (66.7)	3 (42.9)/4 (57.1)	3 (42.9)/4 (57.1)	3 (30)/7 (70)	4 (50)/4 (50)	2 (28.6)/5 (71.4)
Age (mean ± SD)	31.7 ± 6.3	39.5 ± 7.2	38.3 ± 9.5	38.3 ± 7.7	34.3 ± 6.7	38 ± 7
Disease pattern						
Ileocolitis	4 (66.7)	7 (100)	6 (85.7)	9 (90)	6 (75)	6 (85.7)
Ileitis	-	-	1 (14.3)	1 (10)	2 (25)	1 (14.3)
NA	2 (33.3)	-	-	-	-	-
Inflammatory status of the biopsy						
Non-inflamed	5 (83.3)	6 (85.7)	3 (42.9)	6 (60)	4 (50)	4 (57.1)
Inflamed	1 (16.7)	1 (14.3)	4 (57.1)	4 (40)	4 (50)	3 (42.9)
Medication						
Prednisone	1 (16.7)	2 (28.6)	–	2 (20)	–	2 (28.6)
Mesalazine	3 (50)	4 (57.1)	4 (57.1)	4 (40)	4 (50)	2 (28.6)
Sulfasalazine	–	–	–	1 (10)	–	1 (14.3)
Ustekinumab	2 (33.3)	–	2 (28.6)	–	2 (25)	–
Adalimumab	1 (16.7)	–	–	–	–	–
Infliximab	–	–	1 (14.3)	–	–	–
Cyclophosphamide	1 (16.7)	–	–	–	–	–
Metronidazole	–	–	1 (14.3)	–	1 (12.5)	1 (14.3)
Ciprofloxacin	1 (16.7)	–	1 (14.3)	–	1 (12.5)	–

Values are n (%) unless otherwise indicated.

**Table 3:** Clinical characteristics of CD patients included for IHC analysis and PAS-Alcian staining.

	LYZ IHC		PAS-Alcian		KI67	
Total number of patients	11		9		6	
AZA therapy	( −) 6 (54.5)	( +) 5 (45.5)	5 (55.6)	4 (44.4)	( −) 3 (50)	( +) 3 (50)
Disease activity						
Remission	3 (50)	4 (80)	2 (40)	3 (75)	2 (66.7)	2 (66.7)
Active	3 (50)	1 (20)	3 (60)	1 (25)	1 (33.3)	1 (33.3)
Male/female	4 (66.7)/2 (33.3)	2 (40)/3 (60)	4 (80)/1 (20)	2 (50)/2 (50)	3 (100)/0 (0)	3 (100)/0 (0)
Age (mean ± SD)	41.5 ± 15.1	39.8 ± 7.1	45.2 ± 13.5	40.8 ± 7.8	45.7 ± 17	49 ± 16.5
Disease pattern						
Ileocolitis	5 (83.3)	5 (100)	5 (100)	4 (100)	3 (100)	3 (100)
NA	1 (16.7)	–	–	–	–	–
Inflammatory status of the biopsy						
Non inflamed	4 (66.7)	5 (100)	3 (60)	4 (100)	1 (33.3)	2 (66.7)
Inflamed	2 (33.3)	–	2 (40)	–	2 (66.7)	1 (33.3)
Medication						
Prednisone	–	1 (20)	–	1 (25)	–	1 (33.3)
Mesalazine	1 (16.7)	1 (20)	1 (20)	–	1 (33.3)	–
Sulfasalazine	–	1 (20)	–	1 (25)		–
Ustekinumab	4 (66.7)	–	4 (80)	–	2 (66.7)	–
Infliximab	1 (16.7)	–	1 (20)	–	-	–
Adalimumab	1 (16.7)	–	1 (20)	–	1 (33.3)	–
Ciprofloxacin	1 (16.7)	–	–	–	-	–

Values are n (%) unless otherwise indicated.

**Table 4:** Clinical characteristics of CD patients included for ELISA.

Total number of patients	7	
AZA therapy	( −) 4 (57.1)	( +) 3 (42.9)
Disease activity		
Remission	4 (100)	2 (66.7)
Active	0 (0)	1 (33.3)
Male/female	3 (75) / 1(25)	0(0) / 3 (100)
Age (mean ± SD)	36.3 ± 12.3	44.7 ± 18.4
Disease pattern		
Ileocolitis	2 (50)	3 (100)
Ileitis	2 (50)	–
Medication		
Mesalazine	2 (50)	–
TNF-α inhibitor	4 (100)	3 (100)

Values are n (%) unless otherwise indicated.

Immunohistochemical staining of ileal biopsies and ELISA of stool samples further revealed comparable findings to mRNA expression with higher LYZ protein level in patients who received AZA (Fig. 1b–d). Similar to qPCR findings, LYZ levels were comparable between controls and CD patients not treated with AZA (Fig. 1b,c). On the other hand, mucus level was not affected by AZA therapy (Supplementary Fig. S1a,b). The inflammatory status of ileal biopsies was evaluated by histological scoring that involved assessment of epithelial, architectural changes and immune cell infiltration (Supplementary Fig. S1c). The average histological score of AZA-treated group reached 3.2 while it was around 4.2 in CD patients without AZA treatment. Moreover, mRNA levels of several pro-inflammatory cytokines were examined. While levels of *IL1β*, *TNFα* and *IL8* were comparable between AZA-non treated and AZA-treated patients (Supplementary Fig. S1d–f), *IL6* was significantly downregulated in AZA-treated patients (Supplementary Fig. S1g). Due to the finding that IL6 is not expressed by IECs from murine SI organoids (Supplementary Fig. S1h), one may hypothesize that decreased IL6 expression is caused by decreased leukocyte counts, indicated by decreased ileal CD45 mRNA expression, in CD treated patients (Supplementary Fig. S1i).

Proliferation marker, *Ki67* was downregulated on mRNA and protein levels in CD patients independent of AZA treatment compared to controls (Fig. 1e–g). While mRNA *Ki67* was comparable between AZA-treated and control patients (Fig. 1e), IHC revealed increased Ki67 in AZA-treated patients compared to control (Fig. 1f–g).

### Azathioprine suppresses proliferation and promotes IEC differentiation

Beside AZA’s immunomodulatory effect, a novel target was unraveled through boosting PC antimicrobial repertoire. The next set of experiments was dedicated to finding out if this impact is direct or secondary to changes in immune cells. Studies showed that local adaptive immune cells drive stem cell differentiation into secretory specification such as PCs^31,32^. To determine if AZA directly affects the differentiation and functions of PC, small intestinal epithelial cell line, MODE-K cells were examined.

**Figure 2:**
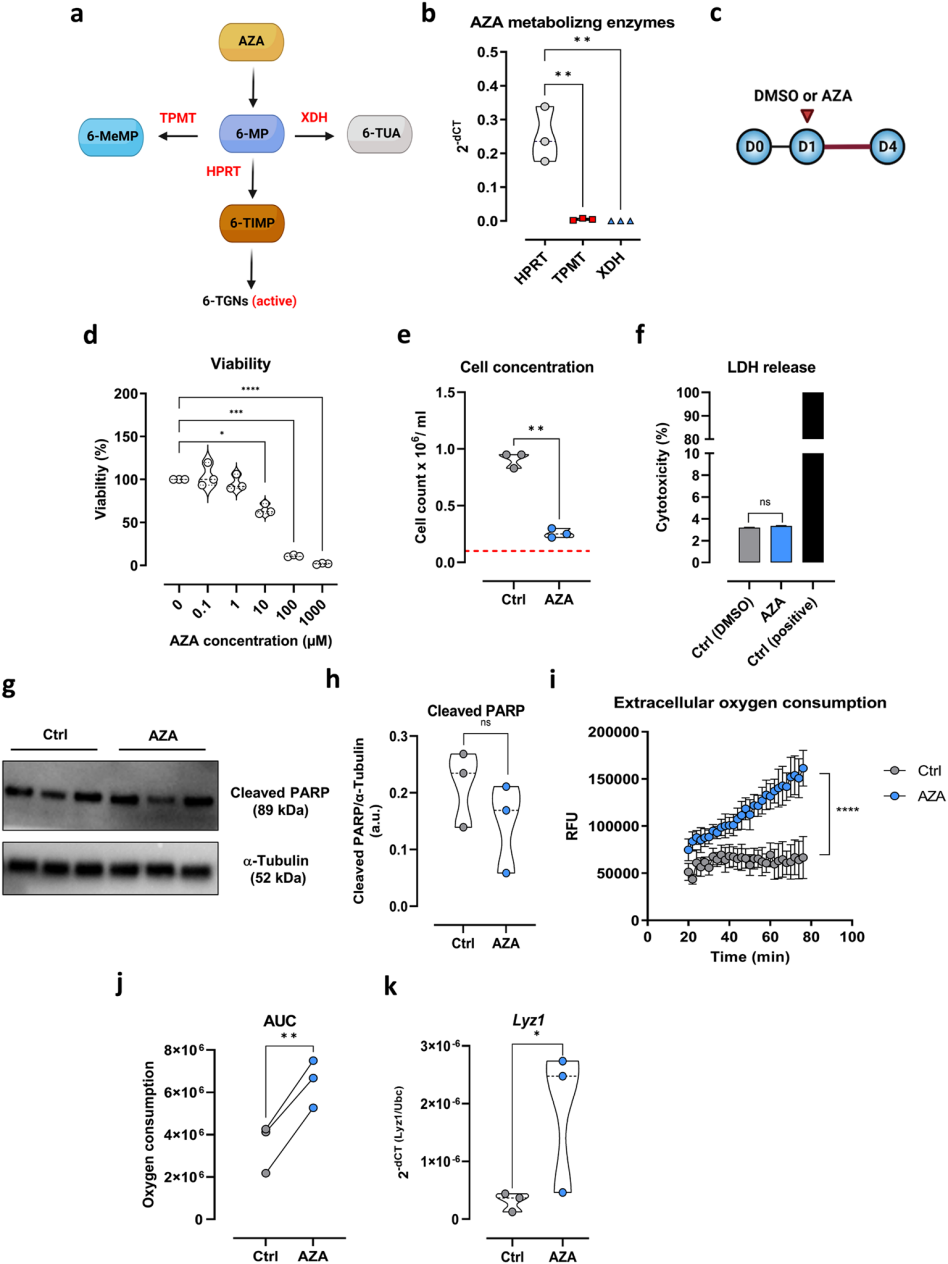
Effect of AZA on the intestinal cell line, MODE-K cells. (**a**) Schematic summary of azathioprine metabolism. (**b**) mRNA expression of *HPRT1*, *TPMT* and *XDH* was measured in MODE-K via qPCR. (**c**) Overnight-incubated MODE-K cells were treated with either AZA or DMSO for three days. (**d**) Cytotoxic effect of AZA was determined in MODE-K treated with various concentrations of AZA (0.1–1000 µM) for three days by neutral red assay. (**e**) Viable cell numbers of DMSO or 1 µM AZA-treated cells were counted by trypan blue exclusion. Red dashed line represents starting seeding density. (**f**) LDH level was measured in the supernatants of AZA and DMSO-treated cells. (**g**) Western blot was performed from whole protein extracts with respective antibodies in cells treated with AZA or DMSO. (**h**) Relative protein expression of cleaved PARP-1 in cells treated with AZA or DMSO. (**i**) Mitochondrial bioenergetics and area under the curve (AUC) (**j**) were measured in MODE-K using MitoXpress Xtra oxygen consumption assay. (**k**) mRNA levels of *Lyz1* were measured in DMSO or AZA-treated cells via qPCR. All experiments were repeated three times. (**b**) one-way ANOVA with Tukey’s multiple comparisons test; (**d**,**i**) one-way ANOVA with Dunnett's multiple comparisons test; (**e**,**f**,**h**,**j**,**k**) paired t test; results are shown as truncated violin plots with median represented as dashed line except (**f**,**i**) mean ± SEM. *p < 0.05, **p < 0.01, ***p < 0.001, ****p < 0.0001. 6-MeMP: 6-methyl mercaptopurine; 6-MP: 6-mercaptopurine; 6-TGNs: 6-thioguanine nucleotides; 6-TIMP: 6-thioinosine monophosphate; 6-TUA: 6-thiouric acid; AZA: azathioprine; HPRT: hypoxanthine phosphoribosyltransferase; LDH: lactate dehydrogenase; LYZ: lysozyme; RFU: relative fluorescence units; TPMT: thiopurine S-methyltransferase; XDH: xanthine dehydrogenase.

First, we examined if MODE-K cells have the capability to metabolize AZA by measuring the mRNA levels of AZA-metabolizing enzymes. Following the swift conversion of AZA to mercaptopurine (6-MP), three primary enzymes—hypoxanthine phosphoribosyltransferase (HPRT), thiopurine S-methyltransferase (TPMT), and xanthine dehydrogenase (XDH)—act upon 6-MP^33^ (Fig. 2a). HPRT plays an important role in the activity of AZA through the formation of 6-MP nucleotides and eventually the active metabolites, thioguanine nucleotides (TGNs)^34^. Alternatively, 6-MP can be converted to inactive metabolites such as 6-thiouric acid (6-TUA) or 6-methylmercaptopurine (6-MeMP) by XDH or TPMT, respectively^35^. MODE-K cells displayed high expression of HPRT and low mRNA levels of *TPMT* and *XDH* which might indicate an increased production of active metabolites of AZA (Fig. 2b).

To specify the working concentration of AZA that does not induce cell death, MODE-K cells were treated with a series of AZA concentrations ranging from 0.1 µM to 1 mM for three days (Fig. 2c). AZA treatment at 1 µM resulted in a slight decrease in MODE-K viability reaching an average of 95%, a significant loss was evident staring from 10 µM and down to 1 mM (Fig. 2d). Next, MODE-K cells were treated with 1 µM AZA for 72 h. Control cells displayed an increase of cell count of around 0.91 × 10^6^ per ml compared to seeded cells, while AZA-treated cells did not, reflected by the cell number of 0.26 × 10^6^ per ml (Fig. 2e). To unravel whether AZA treatment induces cell death in MODE-K cells, we analyzed LDH release into the supernatants by ELISA experiments as well as cleavage of PARP-1^36^, regarded as a characteristic feature of apoptosis by pro-apoptotic caspases, by western blot experiments. Here, we detected unaltered levels of lactate dehydrogenase (LDH) in the supernatant of AZA-treated cells (Fig. 2f) as well as of cleaved PARP in the AZA-treated group compared to the control group (Fig. 2g,h). Together, these data point to a slower proliferation rate rather than induction of cell death or apoptosis in AZA treated SI MODE-K cells.

Additionally, this suppression in cellular proliferation was associated with heightened cellular respiration in AZA-treated cells, further indicating a high cell viability (Fig. 2i,j). Crypt-villus axis exhibits variations of energy metabolism. While not terminally differentiated crypt bottom cells are highly proliferative, fully differentiated cells on the villi display the lowest proliferation capacity^37^. Indeed, a significant increase in *Lyz1,* a marker of differentiated PCs*,* mRNA expression was detected in AZA-treated cells (slowly proliferative) compared to control (highly proliferative) (Fig. 2k).

### Azathioprine improves the differentiation of IECs in 3D SI-organoids

**Figure 3:**
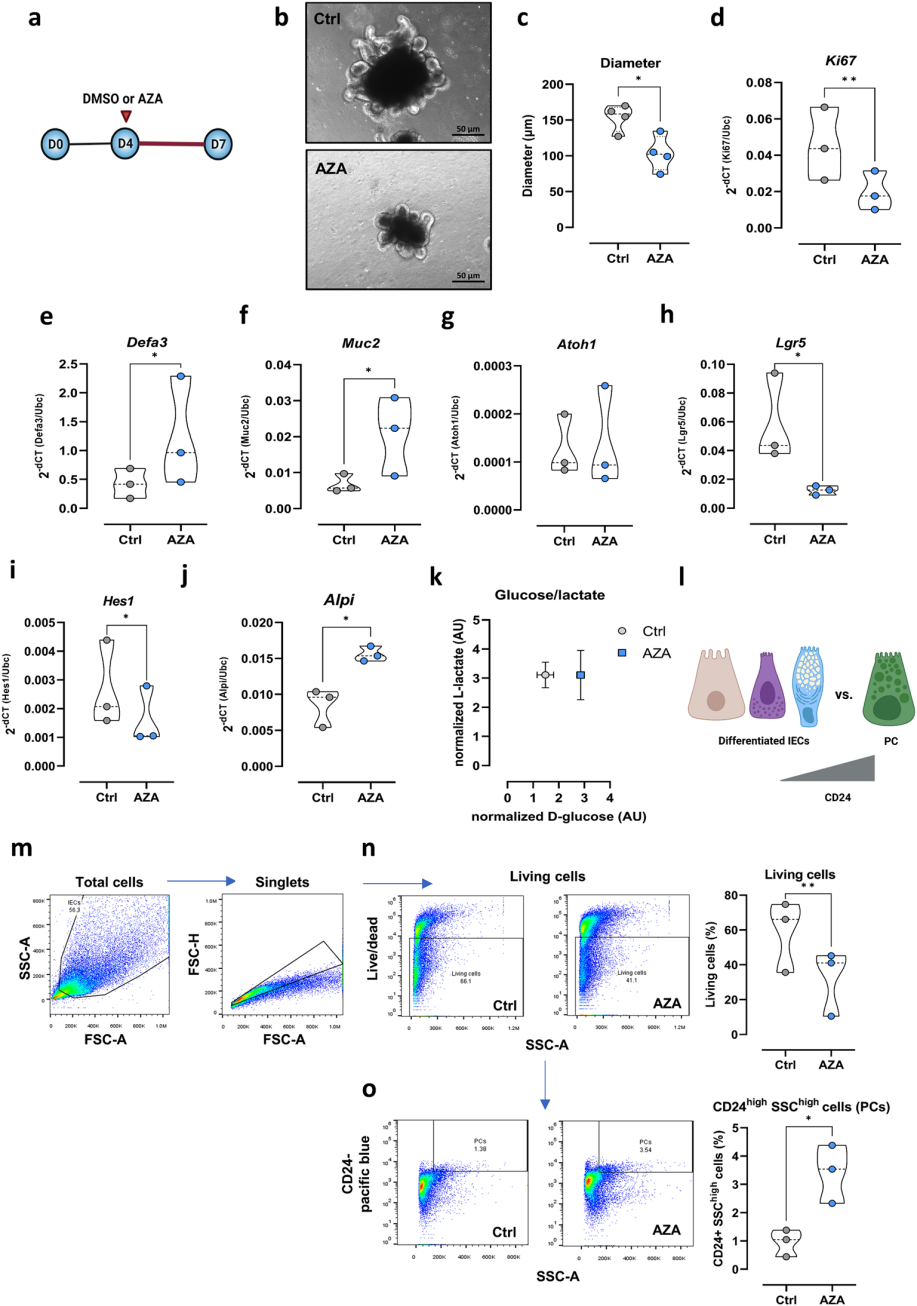
Effect of AZA on small intestinal organoids. (**a**) C57Bl/6 SI organoids were maintained on standard medium for four days. Consequently, DMSO or AZA (1 µM) was introduced on day 4 for three days. (**b**) Microscopic appearance of SI organoids treated with DMSO or AZA. Scale bar = 50 µm (40x). (**c**) Diameter of DMSO and AZA-treated organoids was measured using Zeiss ZEN software. Expression of *Ki67* (**d**), *Defa3* (**e**), *Muc2* (**f**) was measured in ctrl and AZA-treated organoids via qPCR. mRNA expression of *Atoh1* (**g**), *Lgr5* (**h**) and *Hes1* (**i**) and *Alpi* (**j**) in DMSO and AZA-treated organoids. (**k**) Glucose and lactate levels were measured in the supernatants of SI organoids. (**l**) Schematic representation of differences between PCs and other differentiated IECs with regard to CD24 expression. (**m**) Gating strategy of single cell suspension generated from SI organoids treated with DMSO or AZA. Dot plots and percentages of living cells (**n**) and CD24^high^ SSC^high^ (**o**) were measured using Flowjo software. Statistical differences were detected by paired t test. All results are shown as truncated violin plots with median represented as dashed line except (**k**) mean ± SEM. *p < 0.05, **p < 0.01. *Alpi*: intestinal alkaline phosphatase; *Atoh1*: Atonal BHLH Transcription Factor 1; AZA: azathioprine; *Defa3*: alpha-defensin 3; FSC: forward scatter; *Hes1*: Hairy and enhancer of split-1; IEC: intestinal epithelial cell; *Lyz1*: lysozyme 1; *Muc2*: mucin 2; PC: Paneth cell; SSC: side scatter.

In the previous experiments utilizing the small intestinal epithelial cell line MODE-K, AZA demonstrated anti-proliferative activity and the capacity to improve mitochondrial OXPHOS activity that was accompanied by enhanced *Lyz1* expression. To identify the effect of AZA on distinct intestinal epithelial cell subtypes such as stem cells, enterocytes, PCs or goblet cells, we next investigated the effect of AZA treatment on murine 3D-organoid systems generated from small intestinal tissue samples. Microscopically, 3D SI- organoids treated with 1 µM AZA for three days (Fig. 3a) were morphologically distinct from controls (Fig. 3b). Small and rounded structures were associated with AZA treatment (Fig. 3b). Notably, diameter of AZA-treated organoids was around 103 µm while the DMSO-treated group reached 153 µm (Fig. 3c). The microscopic analysis correlated with decreased expression of the cell proliferation marker *Ki67* in AZA-treated organoids compared to control organoids (Fig. 3d). That was validated by IHC experiments on protein level (Supplementary Fig. S2a,b). As already demonstrated in MODE-K cells, AZA treatment of 3D SI-organoids did not induce cell death as revealed by similar LDH levels in the supernatants from both groups, Supplementary Fig. S2c). Moreover, AZA did not trigger apoptosis in IECs, as evidenced by TUNEL staining (Supplementary Fig. S3).

On the other hand, IEC differentiation was heightened in AZA-treated organoids as reflected by significant increase in mRNA expression of the PC marker *Defa3* and the goblet cell marker *Muc2* (Fig. 3e,f). Similarly, IHC experiments indicated numbers of Lyz and PAS-Alcian positive cells to be increased in AZA-treated organoids (Supplementary Fig. S2d–g).

Of note, mRNA expression of the marker for precursors of secretory IECs such as PCs or goblet cells, *Atoh1*, was not regulated by AZA treatment of organoids (Fig. 3g). In contrast, a significant downregulation of the ISC marker *Lgr5* (Fig. 3h) as well as of the enterocyte promoter *Hes1* (Fig. 3i) was seen in AZA-treated organoids by qPCR experiments. Interestingly, absorptive enterocyte marker *Alpi* was significantly upregulated in AZA-treated organoids (Fig. 3j). The observed diminished cell proliferation of AZA-treated 3D SI-organoids was associated with decreased glucose consumption while lactate production remained the same (Fig. 3k), indicating a reduction in aerobic glycolysis activity.

Highly granular PCs highly express CD24, identified by a high side-scatter (SSC^high^) and CD24^high^ in flow cytometry analysis (Fig. 3l)^11,38^. Sorted CD24^high^ SSC^high^ cells exhibited higher mRNA expression of PC markers *Lyz1* and *Defa3* compared to CD24- cells (Supplementary Fig. S4). Flow cytometry analysis utilizing single cell suspension of AZA-treated or control 3D SI-organoids revealed a significant decrease in the IEC viability of AZA-treated organoids (Fig. 3m,n). By gating for living cells with high side scatter (SSC) and high CD24 expression, a significant increase in the percentage of this PC-enriched IEC population was detected in AZA-treated organoids (Fig. 3o).

Together, these results highlight the capacity of AZA to inhibit proliferation but boost IEC differentiation. Importantly, these changes were associated with a reduction in glycolytic activity and an improvement in mitochondrial OXPHOS activity.

### Intact mitochondrial bioenergetics is crucial for proper Paneth cell function

**Figure 4:**
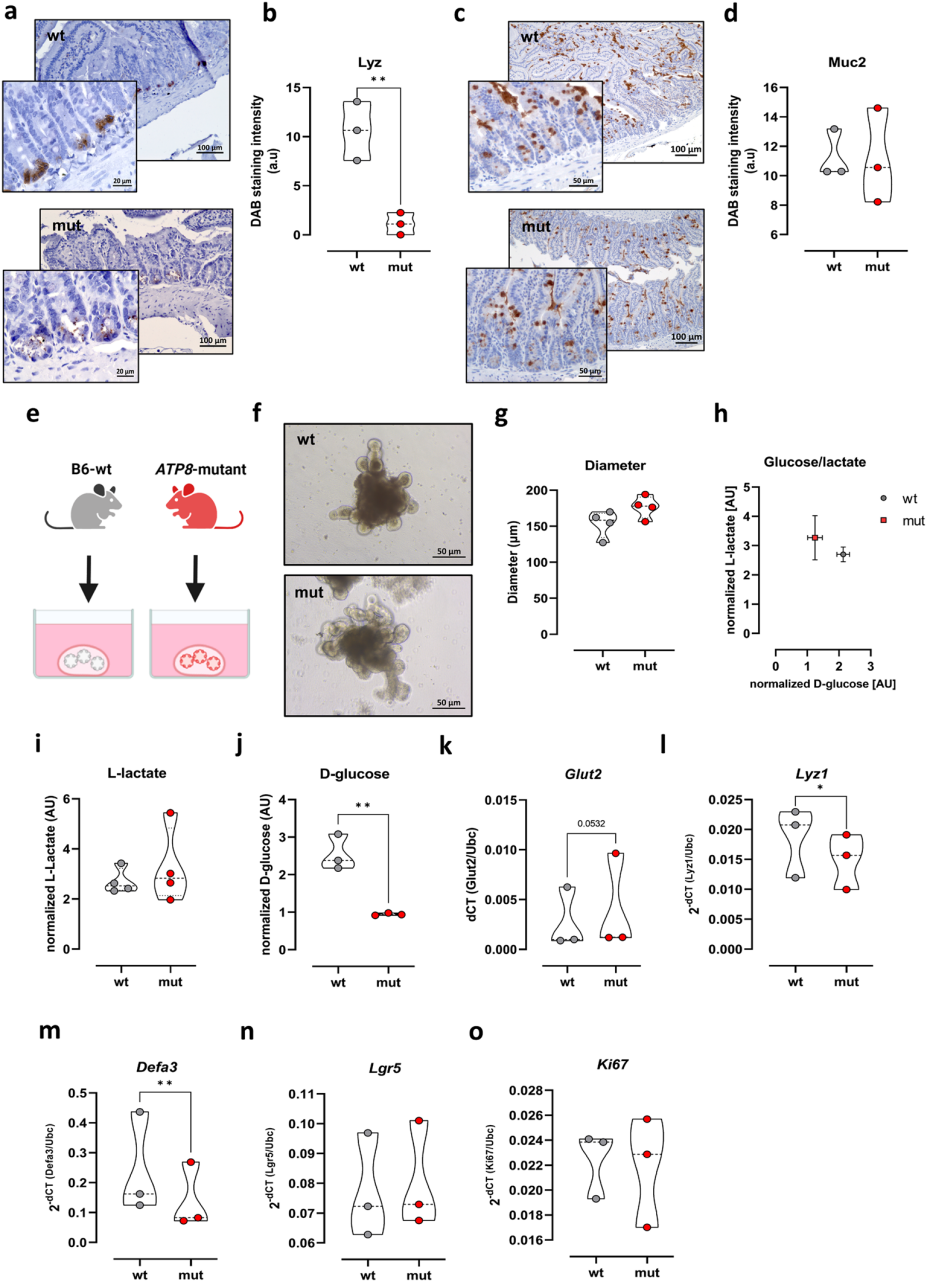
Effect of mitochondrial impairment on PC development. (**a**,**c**) Immunohistochemistry staining of ileal sections from C57BL6/J (wt) and *Atp8* mutant (mut) mice with Lyz and Muc2 antibodies. Scale bar (Lyz) = 100 µm (10x), 20 µm (63x) and Scale bar (Muc2) = 100 µm (10x), 20 µm (63x). Levels of Lyz (**b**) and Muc2 (**d**) in ileal crypts were measured using ImageJ software. (**e**) SI organoids were generated from wt and *Atp8*-mutant mice. (**f**) Microscopic appearance of wt and *Atp8*-mut SI organoids. Scale bar = 50 µm (40x). (**g**) Diameters of wt and *Atp8*-mut SI organoids. (**h**–**j**) Levels of metabolic markers (lactate and glucose) were measured in the supernatants of intestinal organoids and Glut2 mRNA level (**k**) was measured by qPCR. mRNA expression of PC markers; *Lyz1* (**l**) and *Defa3* (**m**) in wt and mut SI organoids. mRNA levels of *Lgr5* (**n**) and *Ki67* (**o**) were measured by qPCR in wt and mut SI organoids. Statistical differences were detected by paired t test. All results are shown as truncated violin plots with median represented as dashed line except (**h**) mean with SD. *p < 0.05, **p < 0.01, ****p < 0.0001. *Defa3*: alpha-defensin 3; *Glut2:* glucose transporter 2; *Lyz1*: lysozyme 1; Muc2: mucin 2.

OXPHOS is the main driver of cell differentiation in the intestinal crypt^16^. Moreover, mitochondrial mutations are associated with CD and PC impairment^15,17^. To investigate the contribution of mitochondrial homeostasis in PCs’ function, we utilized a mouse model with mitochondrial mutation. Several studies demonstrated that cells isolated from mice that carry a mutation in mitochondrial *Atp8* gene displayed loss of OXPHOS function and hence produced less ATP that was compensated by higher glycolysis in comparison to wild-type B6 mice^16,39,40^. In the first set of experiments, we collected ileal tissue samples from these mice and performed IHC analyses that indicated a significant decrease in the numbers of Lyz + cells in ileal crypts (Fig. 4a,b). In contrast, ileal Muc2 levels, a marker for the secretory goblet cells, were not affected by this mitochondrial mutation (Fig. 4c,d).

To functionally validate these findings, we generated 3D organoids from small intestinal tissue samples collected from *Atp8*-mut (mut) or wild-type (wt) control mice with the same nuclear DNA background (Fig. 4e). Morphologically, no difference between mut and wt organoids during a culture period of seven days was observed (Fig. 4f). Moreover, the size of mut organoids was slightly larger than wt control (176.6 vs. 153.5 µm, respectively) (Fig. 4g). In contrast to morphology, mut 3D SI-organoids exhibited an enhanced glycolytic metabolism as demonstrated by a significantly higher glucose consumption and unaffected lactate release into the supernatant (Fig. 4h–j), thereby highlighting the mitochondrial dysfunction in mut 3D SI-organoids. Additionally, an upregulation of *Glut2* mRNA, a glucose transporter mainly present at the basolateral epithelium^41^, was found in mut 3D SI-organoids (Fig. 4k). Further, *Lyz1* and *Defa3* mRNA expression was significantly diminished in mut 3D SI-organoids compared to wt controls (Fig. 4l,m). Of note, these differences were independent of divergent mRNA levels of the intestinal stem cell marker *Lgr5* and the cell proliferation marker *Ki67* (Fig. 4n,o).

Together, these observations emphasize the role of mitochondria function in shaping IEC differentiation and PC development.

### Azathioprine restores aberrant PC functions

**Figure 5:**
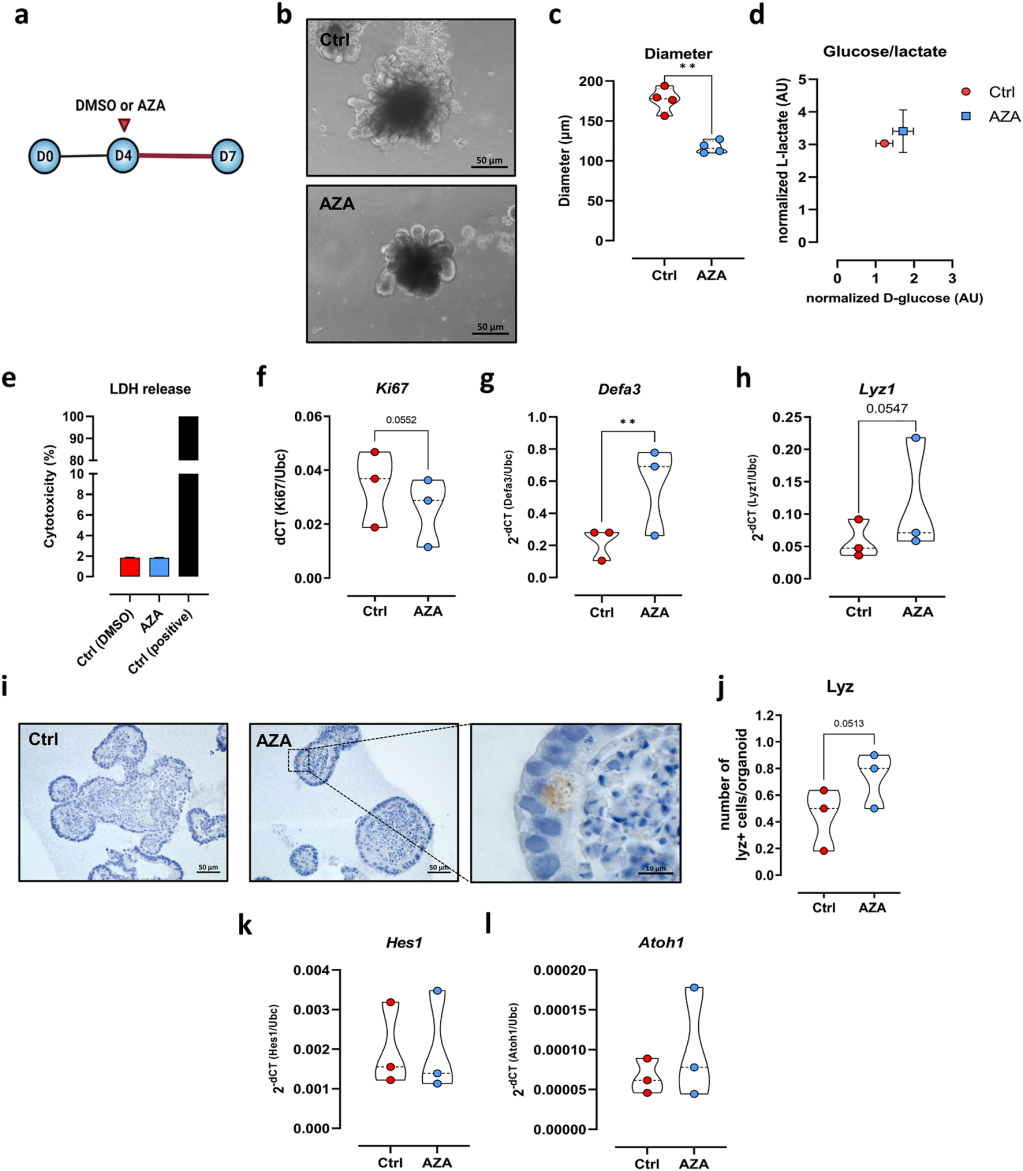
Effect of AZA on intestinal organoids with mitochondrial impairment. (**a**) *Atp8* mutant SI organoids were maintained on standard medium for four days. Consequently, DMSO or AZA was introduced on day 4 at a concentration of 1 µM for three days. (**b**) Microscopic appearance of SI organoids treated with DMSO or AZA. Scale bar = 50 µm (40x). (**c**) Diameter of DMSO and AZA-treated organoids. (**d**) Glucose and lactate levels were measured in the supernatants of SI organoids. (**e**) LDH level was measured in the supernatants of organoids. mRNA expression of Ki67 (**f**), *Defa3* (**g**) and *Lyz1* (**h**) in DMSO and AZA-treated organoids. (**i**) Representative images of organoid sections stained with Lyz antibody. Scale bar = 50 µm (20x), 10 µm (63x). (**j**) Number of Lyz + cells was counted in each organoid. mRNA levels of differentiation markers, *Hes1* (**k**) and *Atoh1* (**l**) were assessed by qPCR. Statistical differences were detected by paired t test. All results are shown as truncated violin plots with median represented as dashed line except (**d**) mean with SEM. **p < 0.01. *Atoh1*: Atonal BHLH Transcription Factor 1; AZA: azathioprine; *Defa3*: alpha-defensin 3; *Hes1*: Hairy and enhancer of split-1; LDH: lactate dehydrogenase.

We next examined if AZA enhancement of mitochondrial bioenergetics could restore PC function in mut 3D SI-organoids. Mutant SI organoids were kept under standard medium for 4 days. Subsequently, medium was exchanged by either DMSO or 1 µM AZA-containing medium and organoids were incubated for additional three days (Fig. 5a). Similar to what was observed with wt organoids (Fig. 4b,c), AZA treatment significantly reduced the size of mut 3D SI-organoids reaching around 117 µm in diameter while DMSO-treated organoids’ diameter was 176 µm (Fig. 5b,c). The reduced organoid growth in the presence of AZA was not accompanied with changes in D-glucose consumption without L-lactate production (Fig. 5d). This indicates the use of glucose to fuel the mitochondrial OXPHOS and not the pentose phosphate pathway in AZA-treated organoids. Neither necrotic cell death nor apoptosis were induced by AZA treatment as investigated by LDH release into the supernatants or TUNEL staining, respectively (Fig. 5e, Supplementary Fig. S3). In line with data presented from MODE-K cells as well as wt SI organoids, Ki67 was downregulated in AZA-treated group (Fig. 5f). On the other hand, mRNA expression of the PC markers, *Defa3 and Lyz1* as well as Lyz + cells were elevated in AZA-treated *Atp8* mut organoids (Fig. 5g–j), while *Atoh1* and *Hes1* expression levels were not affected (Fig. 5k,l).

Deleterious effects on energy supply induced by mitochondrial mutation were resolved by AZA treatment. AZA-treated *Atp8* mutant 3D SI-organoids exhibited elevated numbers of PCs. These findings corroborate the previous observation of enhanced mitochondrial respiration in AZA-treated MODE-K cells.

## Discussion

AZA belongs to a wide spectrum of drugs used in the management of IBD and specifically CD^42^. The immunosuppressive effects of AZA were heavily investigated with slight focus on IECs^20^. Hence, the question of whether and how AZA, an orally administered drug, may impact the proliferation and differentiation of IECs remains understudied. PCs play a crucial role in the maintenance of mucosal immunity^2^. Disrupted PC function and reduced levels of AMPs are associated with ileal CD^43^.

In the current study, we observed that several PC markers were upregulated in AZA-treated CD patients. On the other hand, the only study which addressed the same question found no influence of AZA on mRNA expression of ileal AMPs^44^. While the biopsies used in our study were harvested from patients in active and remission stage, the patients recruited for Kübler et al*.* study were experiencing clinical relapse. Further, the authors investigated the effect of other medications and found no effect on PC markers. It could be argued that these seemingly conflicting results are due to variations in study design. Epithelial proliferation was markedly decreased in CD patients independent of AZA therapy. This finding might be attributable to IBD therapy. Several studies indicated that drugs such as prednisone and mesalazine, which were part of IBD therapy utilized in our cohort study, have direct inhibitory effect on cell proliferation^45,46^. Moreover, ileal CD patients exhibited downregulation of the Wnt transcription factor TCF-4 which is critical for ISC proliferation^47^. On the other hand, inflammatory status of our cohort was found to be comparable between AZA-treated patients and control patients as demonstrated by histological scoring and mRNA expression of various proinflammatory cytokines. One exception was the mRNA level of *IL6* which was downregulated in AZA-treated patients. However, published research as well as analyses in the current study indicate that intraepithelial lymphocytes and enteric neurons, rather than intestinal epithelial cells, are the primary sources of IL6 production^48,49^.

Based on the human ex-vivo findings, it was postulated that AZA specifically targets pathways involved in proliferation and differentiation. This hypothesis was reinforced by in vitro experiments. Using different cell lines and SI organoids, AZA treatment improved differentiation and dampened proliferation. Two independent studies showed similar findings in several epithelial cell lines^27,50^. Anti-proliferative activity of AZA was reported in IEC lines such as human HT-29 cells, rat IEC-6 cells by Schroll et al.^50^. Alpi, a marker of absorptive enterocytes was highly expressed in AZA-treated human colonic T-84 cells^27^. We observed upregulation of several markers of terminally differentiated cells such as *Defa3*, *Muc2* and *Alpi* in AZA-treated SI organoids. Although we did not detect alterations in *Atoh1* expression, our findings revealed a significant downregulation of *Hes1* in AZA-treated organoids. This may suggest that *Atoh1* may exert its prosecretory effect without Hes1 inhibitory influence. *Hes1* is highly expressed in stem cells^51,52^. This might explain the loss of Hes1 which correlated with the downregulation of Lgr5 upon AZA treatment. In another words, final differentiation of enterocytes is boosted by AZA but not the differentiation of stem cells into precursors of absorptive enterocytes.

The impact of AZA on host metabolism is largely undefined, let alone the intestinal metabolism. Riegel et al*.* explored carbohydrate metabolism in rat hepatocytes after intraperitoneal injection of AZA for 2 weeks^53^. They concluded that liver glucose and lactate were comparable to that of their control counterparts. Nonetheless, liver glycogen was significantly decreased in AZA-treated animals. Specifically, glycogen formation was diminished in hepatocytes of AZA-treated group when pyruvate was used as a substrate but not in the presence of the non-essential amino acid, serine. Pyruvate is an important metabolite, sitting at the crossroads of major metabolic pathways namely glycolysis and gluconeogenesis^54^. The aforementioned study is in agreement with our findings, indicating that AZA modulates pathways involved in glucose metabolism.

Mitochondria exert leverage in the stemness and differentiation of IECs. This role is clearly spotted upon mitochondrial impairment associated with IBD. Khaloian et al*.* reported that inducing mitochondrial dysfunction by ISC-specific deletion of mitochondrial chaperone Hsp60 resulted in reduced stemness and the development of abnormal PCs^15^. Further, mitochondrial dysfunction linked to ablation of prohibitin 1 in IECs preceded spontaneous ileitis with abnormalities in PCs functions^17^. Alula et al. demonstrated that PCs of CD patients displayed mitochondrial deformities in comparison with non-IBD controls^55^. These changes were not exclusive to patients with active disease but interestingly involved inactive CD^55^. Accumulation of mutations in mitochondrial DNA eventually results in a wide range of pathological disorders^56^. This is due to the vestigial nature of mitochondrial DNA repair system which make it more susceptible to harmful damages^57^. Grieves et al*.* elucidated how mitochondrial mutations spread in all cells along colonic crypt from one mutated ISC^58^.

Mitochondrial ATP synthase, a rotatory complex, contains 17 protein subunits and consists of a membrane-extrinsic F_1_ catalytic and membrane-embedded F_o_ domains, which are connected by a peripheral and central stalk^59^. Consistent with the previous studies^17,55^, we here demonstrated that a mutation in mitochondrial *Atp8* gene influenced PCs differentiation in both ileum and 3D SI-organoids. Polymorphisms in mitochondrial *ATP8* are linked to gastrointestinal pathologies such as irritable bowel disease^60^. PCs tightly regulate gut microbiota composition through the MyD88 pathway^8,9^. This could explain previous findings when changes were reported in the microbial communities of *Atp8* mutant mice^39^. Accumulating data linked altered host mitochondrial functions and dysbiosis in CD^61,62^.

To compensate for the defect in energy generation, *Atp8* mutant 3D SI-organoids exhibited changes in their metabolic phenotype as demonstrated by increased glucose uptake. Aerobic glycolysis gives rise to pyruvate which is mainly converted to lactate at the expense of entering the tricarboxylic acid cycle (TCA) cycle^63^*.* Another indication of the increased glucose uptake in *Atp8* mutant organoids is the upregulation of the glucose transporter, *Glut2* on mRNA levels. Glut2 was found to be localized at the basolateral side of the epithelium in SI organoids^41^. Despite that Glut2 drives glucose exit into the circulation, the presence of a gradient across the membrane changes the direction to glucose import into the cells^41^. Given that organoid is a closed system, it is assumed the existence of a gradient that activates Glut2.

Contrary to what we observed with the boosted mitochondrial respiration, the majority of publications state that AZA causes hepatic mitochondrial injury and ATP depletion^64,65^. One explanation for this toxic effect is the high AZA concentration used in these studies. Mitochondrial injury was observed with a dosage of above 100 µM which exceeds the standard therapeutic doses (1.5–2.5 mg/kg body weight). Exceptionally, Khare et al*.* reported a decrease in intracellular reactive oxygen species (ROS) in AZA-treated intestinal cells exposed to mitochondrial stress^27^. In Khare et al*.* and the present study, a maximum concentration of 10 µM was used. Taken together, these reported variations might be due to cell-specific effect and targeting of highly proliferative cells.

One of the major challenges in our study is the collection of human samples. The onset and duration of AZA therapy could not be verified in all patients. Additionally, since IBD management usually includes combination therapy, it can be argued that the ameliorating effects observed in AZA-treated patients might be due to synergistic effect. Nonetheless, the real impact of AZA on PCs was clearly evident when in vitro assessment was performed. Duration of AZA therapy usually takes months, but the positive effects were observed after only three days in the present study. A longer treatment period that can mimic in vivo scenario could not be implemented due to the drawbacks associated with cell culture models. Therefore, an animal study is still needed to validate these findings but is out of scope of the current study. Unlike goblet cells, the overall abundance of PCs was low in SI organoids regardless of treatment. This is physiologically similar to our in vivo observations. In the ileum, the number of PCs typically ranges from 2 to 4 cells per crypt, whereas the count of goblet cells is significantly higher, ranging from 6 to 12 cells per crypt.

**Figure 6:**
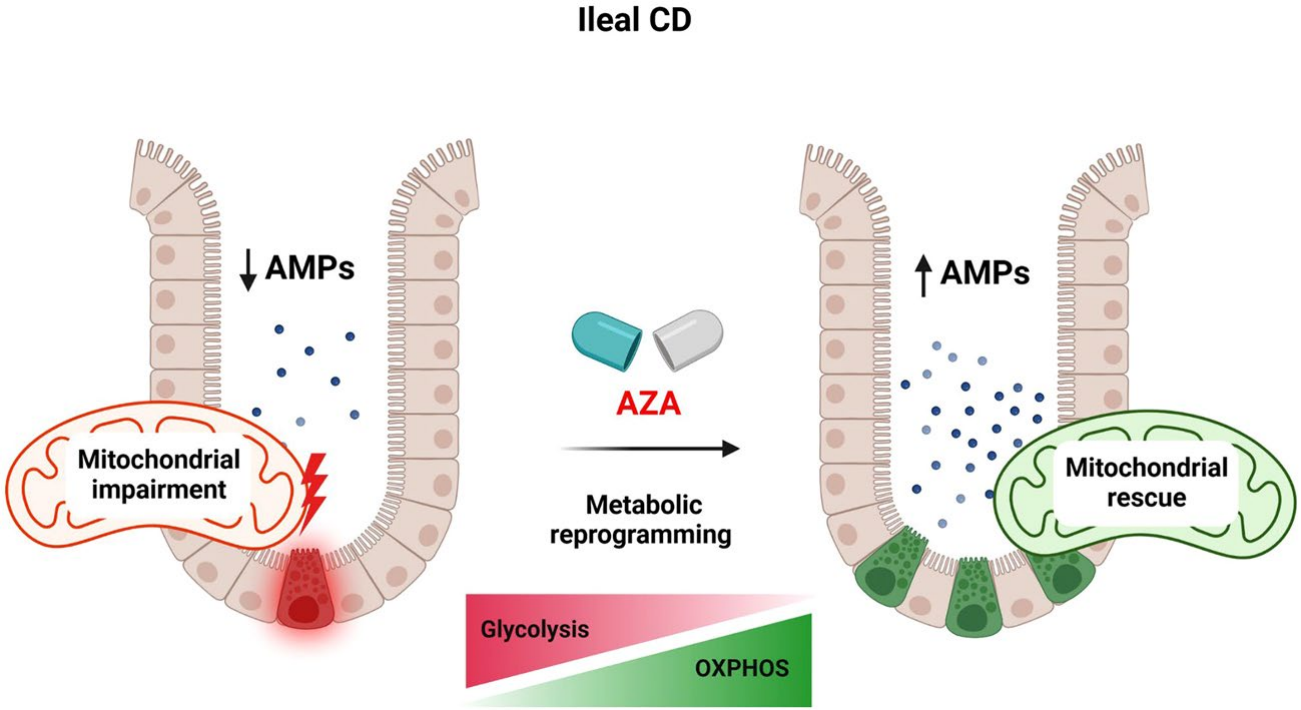
A schematic description of the impact of AZA on intestinal metabolism and PC function. Designed with Biorender.com.

In conclusion, mitochondria are essential for maintaining PC development and function (Fig. 6). Metabolic alterations define cell fate within the intestinal crypt. In mt-*Atp8* mutant mice, the steady-state metabolism is shifted towards glycolysis at the expense of mitochondrial OXPHOS. As a consequence, levels of AMPs were decreased in the ileal compartment of these mice which is likely behind the changes observed in their gut microbiota. CD patients exhibited similar characteristics which have been linked to mitochondrial impairment. Interestingly, AZA was found to reverse this impairment through metabolic modulation. AZA induced changes in IECs with specific effect towards PCs by targeting mitochondrial OXPHOS and glycolysis pathways.

This study clearly revealed an unexplored aspect of AZA mechanism of action. This will open the way to a better refinement of CD therapy according to patients’ needs. In that respect, further investigations are needed to pinpoint the pathways targeted by AZA in IECs. Nam et al*.* identified novel metabolic targets of AZA in patients with glioblastoma^66^. Downregulation of proteins involved in cholesterol biosynthesis, was observed in cells treated with AZA^66^. Various studies reported that dysregulated lipid metabolism contributed to PC impairment^67,68^. Moreover, the active metabolites of AZA are diverse, and the question still remains whether these molecules possess unique features and more studies are required to elucidate if the AZA-induced changes observed in PCs are reflected on gut microbiota and lipid metabolism.

## Methods

### Study cohort

Tissue biopsies from the terminal ileum were collected by endoscopy from hospitalized healthy controls and CD patients from the medical department I, University Hospital Schleswig–Holstein Campus Lübeck and Clinic and Polyclinic for Internal Medicine I, University Hospital Regensburg. Stool samples were obtained from the University Hospital Schleswig–Holstein Campus Lübeck and the University Hospital Münster, North Rhine-Westphalia, Germany. Biopsies were either freshly frozen in liquid nitrogen and stored at − 80 °C or fixed with 4% PFA for histological analysis. Stool samples were frozen immediately after collection at − 80 °C. Clinical characteristics of patients included in this cohort are listed in Tables 1, 2, 3, 4. Samples were collected upon receiving informed consent from each patient. Protocols were approved by the ethics committees of the Lübeck University Hospital (0-073; 03-043; AZ 13/084A; AZ 05-112), the University Hospital Münster (AZ 2016-305-b-S) and the University Hospital Regensburg (21-2390-101 and 22-104). All experiments were performed in accordance with relevant guidelines and regulations.

### Animal models

Adult C57BL/6J and *Atp8*-mut mice (10–13 weeks) were sacrificed for organ collection in accordance with the guidelines approved by the ethics committee, Schleswig–Holstein, Germanyn (C57BL/6FVB: V 242—63560/2017 (5–1/18) and ARRIVE guidelines. All mice were housed under specific pathogen-free conditions at a 12 h light/dark cycle and had access to food and water ad libitum. The conplastic strain C57BL/6J-mt^FVB/NJ^ (*Atp8*-mut) was generated as described previously^69^ and was maintained by repeated backcrossing of female conplastic offspring with male C57BL/6J mice.

### Cell lines

MODE-K (murine small intestinal cell line, generated based on the method described by Vidal et al.^70^, kindly provided by Prof. Mathias Hornef, Institute of Medical Microbiology, University Hospital RWTH Aachen, Germany) were cultured in DMEM medium supplemented with 10% (v/v) heat-inactivated FCS, 0.000001% (v/v) β-mercaptoethanol, 100 U/ml penicillin, and 100 µg/ml streptomycin. Cells were incubated at 37 °C and 5% CO_2_. Cells were passaged twice weekly and checked monthly for mycoplasma contaminants.

To determine the cytotoxic effects of AZA, MODE-K cells were treated with different concentrations of AZA, ranging between 0.1 and 1000 µM for 72 h. DMSO concentration was between 0.0001 and 1% (v/v). Cell viability was determined by neutral red assay. MODE-K cells were treated with 1 µM AZA (Sigma-Aldrich, St Louis, MO) or DMSO (0.001%, v/v) for 72 h after 24 h incubation with no treatment. Cells were either seeded in 96-well plates (5000 cells/well) or 24-well plates at a density of 100,000 cells/well.

### Intestinal organoid culture

Intestinal crypts were generated from the small intestine of age and sex matched *Atp8*-mutant and wild type C57BL6/J mice using the protocol provided by STEMCELL Technologies. In short, small intestinal fragments from both genotypes were washed several times in cold 1 × PBS and then incubated in 25 mL of Gentle Cell Dissociation Reagent (GCDR) at room temperature for 15 min. Several fractions were generated by passing supernatants through 70 µm filters. The quality of each fraction was evaluated using an inverted microscope and fractions with high crypts numbers were selected and centrifuged at 290 × *g* and 4 °C for 5 min. Crypts were then resuspended in cold DMEM/F-12 medium, counted and aliquoted into 15 mL tubes in volumes containing 500–3000 crypts and centrifuged at 200 × *g* and 4 °C for 5 min. Crypts were resuspended in a 150 µl:150 µl mixture of complete IntestiCult Organoid Growth Medium and Geltrex reduced growth factor basement membrane matrix or Corning Matrigel Growth Factor Reduced Basement Membrane Matrix. Domes were formed upon adding 50 µL per well of the suspension into pre-warmed 24-well plates. Consequently, domes were solidified by incubating the plate at 37°C for 10 min. Domes were overlaid with IntestiCult Organoid Growth Medium and incubated at 37 °C, 5% CO_2_ for 7 days with medium changes every 2–3 days.

B6 and *Atp8*-mutant SI organoids were maintained on IntestiCult Organoid Growth Medium for 4 days. Consequently, medium was exchanged with fresh medium supplemented with DMSO (0.001%, v/v) or AZA (1 µM). Organoids were incubated 37 °C, 5% CO_2_ for three days. Supernatants were collected and organoids were harvested for RNA isolation, flow cytometry analysis or histological examination.

### RNA extraction, cDNA synthesis and qPCR

Total RNA was isolated from tissue biopsies, cell pellets or organoids using innuPREP RNA Mini Kit according to the manufacturer's protocol. Samples were homogenized in lysis buffer with the addition of 1% (v/v) β-mercaptoethanol. RNA column was incubated with 4 units DNase, 4 µl 10 × DNase reaction buffer and 32 µl nuclease free H_2_O for 20 min at RT. RNA was eluted with 40 µl nuclease free H_2_O and RNA concentration and quality were determined by Microplate Reader SpectraMax® iD3. (Molecular Devices, California, USA). Samples were stored at -80 °C until cDNA synthesis.

For cDNA synthesis, Reaction mixture for cDNA synthesis consisted of the following: 500–1000 ng RNA, 100 pmol Oligo(dt)18, dNTP Mix (0.2 mM each), 20 U RiboLock RNase inhibitor, and 200 U RevertAid H Minus reverse transcriptase. The mixture was incubated at 42 °C for 60 min followed by 70 °C for 10 min. Samples were diluted 1:5 (v/v) with nuclease free H_2_O and stored at − 20 °C until further analysis.

Target genes were amplified on StepOne real-time system by utilizing the Perfecta SYBR Green Supermix and respective forward and reverse primers (Table 5). Cycling conditions were in the following order using StepOnePlus Real-Time PCR System: an initial denaturation of 95 °C for 5 min, followed by 40 cycles of 95 °C for 45s, 55 °C for 30s and elongation at 72 °C for 30s. Consequently, melting curves were obtained by the following cycling conditions: 95 °C for 15 s, 60 °C for 20 s and 95°C for 15 s. Ct-Values of targets were acquired via the StepOne system software and normalized to β-actin or Ubc that served as an internal housekeeping transcript via the 2^−dCT^ or 2^−ddCT^ algorithm.

**Table 5:** List of oligonucleotides.

Target gene (Human)	Forward primer (5′–3′)	Reverse primer (5′–3′)
*ACTB*	ACATCCGCAAAGACCTGTACG	TTGCTGATCCACATCTGCTGG
*DEFA5*	GGCTACAACCCAGAAGCAGT	CAGCGACAGCAGAGTCTGTA
*DEFA6*	CACCATCCTCACTGCTGTTCT	GCAATGGCAAGTGAAAGCCC
*KI67*	CCTGCTTGTTTGGAAGGG	GCTGGCTCCTGTTCACGTAT
*IL1β*	ATGGCAGAAGTACCTGAGCTCG	CATCGTGCACATAAGCCTCGT
*IL6*	GTGCCAGTATTCCCAGGAGT	TGCAAGATTCCACAACCCTG
*IL8*	TGAGAGTGATTGAGAGTGGACCA	TCAGCCCTCTTCAAAAACTTCTCC
*LYZ*	GCCTAGCAAACTGGATGTGT	ATGCCTTGTGGATCACGGAC
*TNF-α*	ATGAGCACTGAAAGCATG	CAGGGCAATGATCCCAAA

Target gene (Mouse)	Forward primer (5′–3′)	Reverse primer (5′–3′)
*Atoh1*	GTGGGGTTGTAGTGGACGAG	GTTGCTCTCCGACATTGGG
*Defa3*	CCAGGCTGATCCTATCCAAA	GTCCCATTCATGCGTTCTCT
*Glut2*	GTCCAGAAAGCCCCAGATACC	GTGACATCCTCAGTTCCTCTTAG
*Hes1*	CAAACCAAAGACGGCCTCT	GTCACCTCGTTCATGCACTC
*Hprt1*	TCCCAGCGTCGTGATTAGC	TTATGTCCCCCGTTGACTGA
*Ki67*	CCTGCCCGACCCTACAAAAT	TTGCTCACACTCGATGCAGT
*Lgr5*	CGGCAACAGTGTGGACGACCT	GCGAGCACTGCACCGAGTGA
*Lyz1*	GCCAAGGTCTACAATCGTTGTGAGTTG	CAGTCAGCCAGCTTGACACCACG
*Muc2*	GCTGACGAGTGGTTGGTGAATG	GATGAGGTGGCAGACAGGAGAC
*Tpmt*	CAGAGTGGACTGCGAGTGTT	CATAAAATGGTGGGCCTGCG
*Ubc*	GAGCCCAGTGTTACCACCAA	CACACCCAAGAACAAGCACA
*Xdh*	CCTTAGAAGAAAGTTGGGGCTG	CTGTACAGCGGCAGAGGTTT

Primers were purchased from Metabion international AG (Planegg, Germany).

### Extracellular oxygen consumption assay

Respiration rates of DMSO or AZA-treated cells were measured using MitoXpress Xtra Oxygen Consumption Assay (Agilent, California, USA). After three days of treatment with DMSO or AZA, 80.000 cells/well were seeded in 96-well plate in triplicates. Fluorescence was kinetically measured according to manufacturer’s instructions.

### D-Glucose assay

D-glucose level was measured in cell and organoid culture supernatants (1:50 v/v) using the Fluitest GLU kit (Analyticon Diagnostics, Lichtenfels, Germany) according to manufacturer’s instructions.

### L-lactate assay

L-lactate level was measured in cell and organoid culture supernatants (1:10 v/v) using the kit from Megazyme according to manufacturer’s instructions.

### Lactate dehydrogenase assay

Lactate dehydrogenase release was measured in cell and organoid culture supernatants using CyQUANT™ LDH Cytotoxicity Assay (Invitrogen) according to manufacturer’s instructions.

### Human lysozyme ELISA

Lysozyme concentration in protein extracts from human stool samples (1:100) was measured using Human Lysozyme ELISA Kit (Abcam, ab267798, Cambridge, UK). Values were normalized to total protein concentration.

### Histology and microscopy analyses

Immunohistochemical staining was performed utilizing paraformaldehyde-fixed and paraffin-embedded tissue Sects. (2–4 µm). After deparaffinization, rehydration, endogenous peroxidase blockage, and antigen retrieval, specific primary antibodies or isotype control antibodies (Table 6) diluted in 2% (w/v) BSA in 1 × PBS were applied to tissue slides and incubated in wet chambers for 45 min at RT. After washing with 1 × PBS, slides were incubated with HRP-conjugated secondary antibodies or HRP-labelled polymers for 45 min at RT in wet chambers. Slides were incubated for 10 min at RT with DAB substrate solution. Finally, samples were counterstained with Mayer’s hemalum solution and mounted with Aquatex® solution.

**Table 6:** List of antibodies.

Primary antibodies	Host Species	Company	Working concentration
α-Tubulin	Rabbit	Cell Signalling (Danvers, Massachusetts, USA)	1:1000 (WB)
Ki67	Rat	Biolegend (San Diego, California, United states)	1:500 (IHC)
Ki67(clone MIB-1)	Mouse	Agilent (Santa Clara, California, United States)	1:500 (IHC)
Lysozyme (GT1123)	Rabbit	Genetex (Irvine, California, United States)	1:500 (IHC)
Mucin 2 (C3)	Rabbit	Genetex (Irvine, California, United States)	1:500 (IHC)
Cleaved PARP (Asp214)	Rabbit	Cell Signalling (Danvers, Massachusetts, USA)	1:1000 (WB)
Isotype control	Goat	R&D Systems (Minneapolis, Minnesota, USA)	
Isotype control	Mouse	Biotech (Onsala, Sweden)	

Secondary antibodies/labelled polymers		Company	Working concentration
Anti-mouse IgG HRP		Cell Signalling (Danvers, Massachusetts, USA)	1:400 (IHC)
Anti-rabbit IgG HRP		Cell Signalling (Danvers, Massachusetts, USA)	1:400 (IHC)
Anti-goat IgG HRP		Agilent (Santa Clara, California, USA)	1 µg/ml (IHC);

For PAS-Alcian staining, deparaffinized sections were incubated with Alcian blue for 30 min, followed by 1% (v/v) periodic acid for 10 min and eventually with Schiff's reagent for 15 min. Tissue slides were counterstained with Mayer’s hemalum solution. Staining was evaluated using Axio Scope.A1 microscope and analyzed using the color deconvolution plugin in ImageJ software.

TUNEL staining was performed to detect apoptotic cells using ApopTag® Fluorescein in situ apoptosis detection Kit (Millipore) according to manufacturer’s instructions.

The histopathologic scoring system comprised of three parameters, epithelial damage, architectural distortion and immune cell infiltration (0–3). Each aspect was assessed using a four-point rating system: absent (0), mild (1), moderate (2), or severe (3).

### Neutral red assay

Cell viability was measured by the neutral red uptake assay using the protocol developed by Repetto et al.^71^. In brief, neutral red medium was centrifuged for 10 min at 400 × *g* to remove any precipitated dye crystals. Medium was removed from cells and 100 µl of neutral red medium/well was added. Plate was incubated at 37 °C, 5% CO2 for 2 h. Next, neutral red medium was discarded, and wells were washed with 150 µl 1 × PBS. After decanting PBS, 150 µl of neutral red destain solution was added and plate was placed on a microtiter plate shaker for 10 min at RT. OD was measured at 540 nm against a reference wavelength of 690 nm on a spectrophotometer.

### Western blot

Proteins were extracted from samples by resuspension in denaturing lysis buffer with the addition of protease, phosphatase II and III inhibitors (Sigma-Aldrich). Concentration was quantified according to Bradford by rotiquant assay. A mixture of protein samples (15–40 µg) and sodium dodecyl sulfate (SDS) with containing 10% β-mercaptoethanol, was loaded onto a precast polyacrylamide gel. Proteins were separated by gel electrophoresis and blotted to polyvinylidene difluoride (PVDF) membranes according to standard protocols. After blotting, membranes were blocked in 5% (w/v) non-fat milk in Tween-TBS (T-TBS) for 1 h at RT. Diluted primary antibodies (Table 6) in 5% non-fat milk or 5% BSA in T-TBS were applied to membranes and incubated at 4 °C overnight. Membranes were washed two times for 20 min in T-TBS buffer. Membranes were incubated with respective horse-radish peroxidase (HRP) conjugated secondary antibodies (Table 6) for 1 h at RT. Another 2 rounds of washing with T-TBS were performed. Finally, HRP substrate solution was evenly distributed on membranes and chemiluminescence was detected under UV light in the ChemiDocTM XRS + imaging system using the ImageLab™ software. Intensity of chemiluminescence was quantified via the Fiji plugin of ImageJ software and expression levels of proteins of interest were normalized to housekeeper proteins.

### Flow cytometry and cell sorting of intestinal organoids

To generate single cell suspension, organoids were collected in 15 ml Falcon tubes and incubated with cold GCDR on ice for 10 min with frequent tapping. Then, organoids were pelleted by centrifuging for 5 min at 200 × g at 4 °C. Pellet was resuspended in 5 ml of cold DMEM/F12 + 15 mM HEPES. Samples were centrifuged for 5 min at 200 × *g* at 4 °C and warm Trypsin–EDTA (0.05%) was added to the pellets and vigorously resuspended. Cell suspension was incubated at 37 °C for 5–8 min. Samples were briefly vortexed and DMEM/F12 + 10% (v/v) FCS was added and vigorously mixed. Subsequently, cells were pelleted and washed with 1% (w/v) BSA in 1 × PBS. Cells were pelleted and incubated with viability dye efluor 780 (1:1,500) and pacific blue anti-mouse CD24 (1:100) or pacific blue isotype control in 1% (w/v) BSA/PBS on ice in the dark for 20 min. Cells were washed in 1% (w/v) BSA in 1 × PBS and pellets were eventually resuspended in 1% (w/v) BSA in 1 × PBS. Events were measured using Attune NxT flow cytometer (Thermo Fisher Scientific Inc, Massachusetts, USA) and analyzed by FlowJo software. Gates were determined based on unstained and isotype controls (Supplementary Fig. S5).

For cell sorting, BD FACSAria™ III Cell Sorter (BD Biosciences, New Jersey, USA) was used to sort CD24^high^ SSC^high^ and CD24- cells from single-cell suspensions derived from intestinal organoids.

### Statistical analysis

Statistical analysis was performed with GraphPad Prism version 9 (San Diego, CA, USA). ROUT test was used to identify outliers with Q value set at 1%. Data were tested for normal distribution using D’Agostino & Pearson test. Statistical differences of normally distributed data were assessed by t-test while Mann–Whitney U test was used for not-normally distributed data. The specific statistical test employed for each graph is outlined in the figure legend. Differences between groups were considered to be significant at a *p*-value of * < 0.05, ** < 0.01, *** < 0.001, or **** < 0.0001.

## Data Availability

All data analyzed during this study are included in this published article.

## Supplementary Figure

Supplementary Figures. Supplementary Information.
