# Supplementary material for: Azathioprine promotes intestinal epithelial cell differentiation into Paneth cells and alleviates ileal Crohn’s disease severity
Source: Sci Rep. 2024 Jun 5;14:12879. doi: 10.1038/s41598-024-63730-4 (PMC11153537; doi:10.1038/s41598-024-63730-4)
Supplement: Supplementary file 1 — Supplementary Figures. [file 41598_2024_63730_MOESM1_ESM.pdf]

# Supplementary Figure Legends

**Figure S1**. **Effect of AZA on CD patients.** (a) Ileal sections from CD patients were stained with PAS-Alcian to measure mucin levels. Scale bar = 100 µm (10x). (b) Intensity of the staining per crypt was measured using ImageJ software. (c) inflammation severity of ileal biopsies from CD patients was evaluated by histological scoring. qPCR was performed to measure mRNA levels of *IL1b* (d)*, TNF-α* (e)*, IL8* (f) and *IL6* (g) in ileal biopsies from CD patients. (h) mRNA expression of *IL6* in murine SI organoids. (i) Ileal mRNA expression of CD45 in CD patients. Mann-Whitney test was performed. Results are shown as truncated violin plots with median represented as dashed line. **p* < 0.05 CD: non-AZA treated CD patients; CD-A: azathioprine-treated CD patients

**Figure S2**. **Effect of AZA on B6 SI organoids.** (a) Ki67 level was assessed by IHC in B6 SI organoids treated with either DMSO (ctrl) or AZA for three days. Scale bar: 50 µm (20x). (b) Numbers of Ki67+ cells were counted in SI organoids. (c) LDH level was measured in the supernatants of AZA and DMSO-treated organoids. (d) IHC was performed to evaluate Lyz expression in organoids treated with DMSO or AZA. Scale bar: 20 µm (63x) (e) Average number of Lyz+ cells in SI organoids. (f) Representative images of PAS-Alcian staining with respective quantification (g). Scale bar: 20 µm (20x). Paired t test was performed. Data are shown as mean ± SEM. ***p* < 0.01. AZA: azathioprine; LDH: lactate dehydrogenase; Lyz: lysozyme; PAS: periodic acid Schiff

**Figure S3**. **Effect of AZA on apoptosis of IECs.** Representative images of tissue sections from B6 (wt) and Atp8 mutant (mut) SI organoids, treated with either DMSO (ctrl) or AZA, were stained with TUNEL (green) and DAPI (blue). A representative murine colonic tissue section was used as a positive control. Scale bar: 50 µm (20x).

**Figure S4**. **mRNA levels of PC markers in CD24$^{high}$ SSC$^{high}$.** (a) Gating strategy of single cell suspension derived from SI organoids. (b) qPCR was performed to measure levels of *Lyz1* and *Defa3*in sorted CD24$^{high}$ SSC$^{high}$ and CD24- cells. **$p < 0.01$. Results are shown as mean ± SD. *Defa3*: alpha-defensin 3; FSC: forward scatter; IEC: intestinal epithelial cell; *Lyz1*: lysozyme; SSC: side scatter.

**Figure S5**. **Flow cytometry controls.** The gating strategy of live cells was established by incubating single cell suspension from SI organoids for 20 min in the absence (a), or in the presence (b) of viability dye. Moreover, cells were either unstained (c) or stained for CD24 (d) or its isotype (e) in the presence of viability dye to exclude dead cells.

# Figure S1

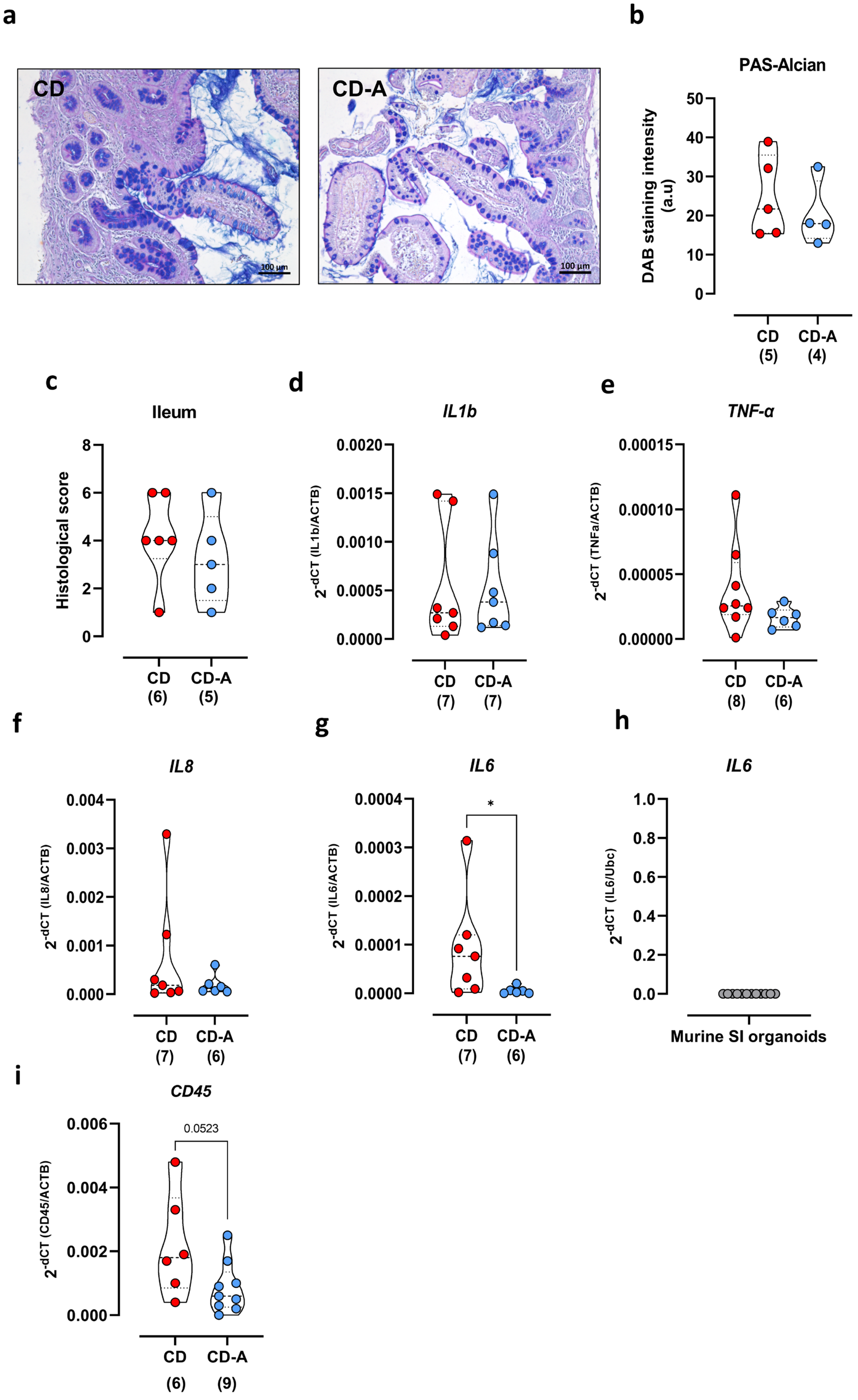

a
CD
CD-A
100 µm
100 µm
b
PAS-Alcian
DAB staining intensity (a.u)
50
40
30
20
10
0
CD (5)
CD-A (4)
c
Ileum
Histological score
8
6
4
2
0
CD (6)
CD-A (5)
d
IL1b
2-dCT (IL1b/ACTB)
0.0020
0.0015
0.0010
0.0005
0.0000
CD (7)
CD-A (7)
e
TNF-α
2-dCT (TNFa/ACTB)
0.00015
0.00010
0.00005
0.00000
CD (8)
CD-A (6)
f
IL8
2-dCT (IL8/ACTB)
0.004
0.003
0.002
0.001
0.000
CD (7)
CD-A (6)
g
IL6
*
2-dCT (IL6/ACTB)
0.0004
0.0003
0.0002
0.0001
0.0000
CD (7)
CD-A (6)
h
IL6
2-dCT (IL6/Ubc)
1.0
0.8
0.6
0.4
0.2
0.0
Murine SI organoids
i
CD45
0.0523
2-dCT (CD45/ACTB)
0.006
0.004
0.002
0.000
CD (6)
CD-A (9)

# Figure S2

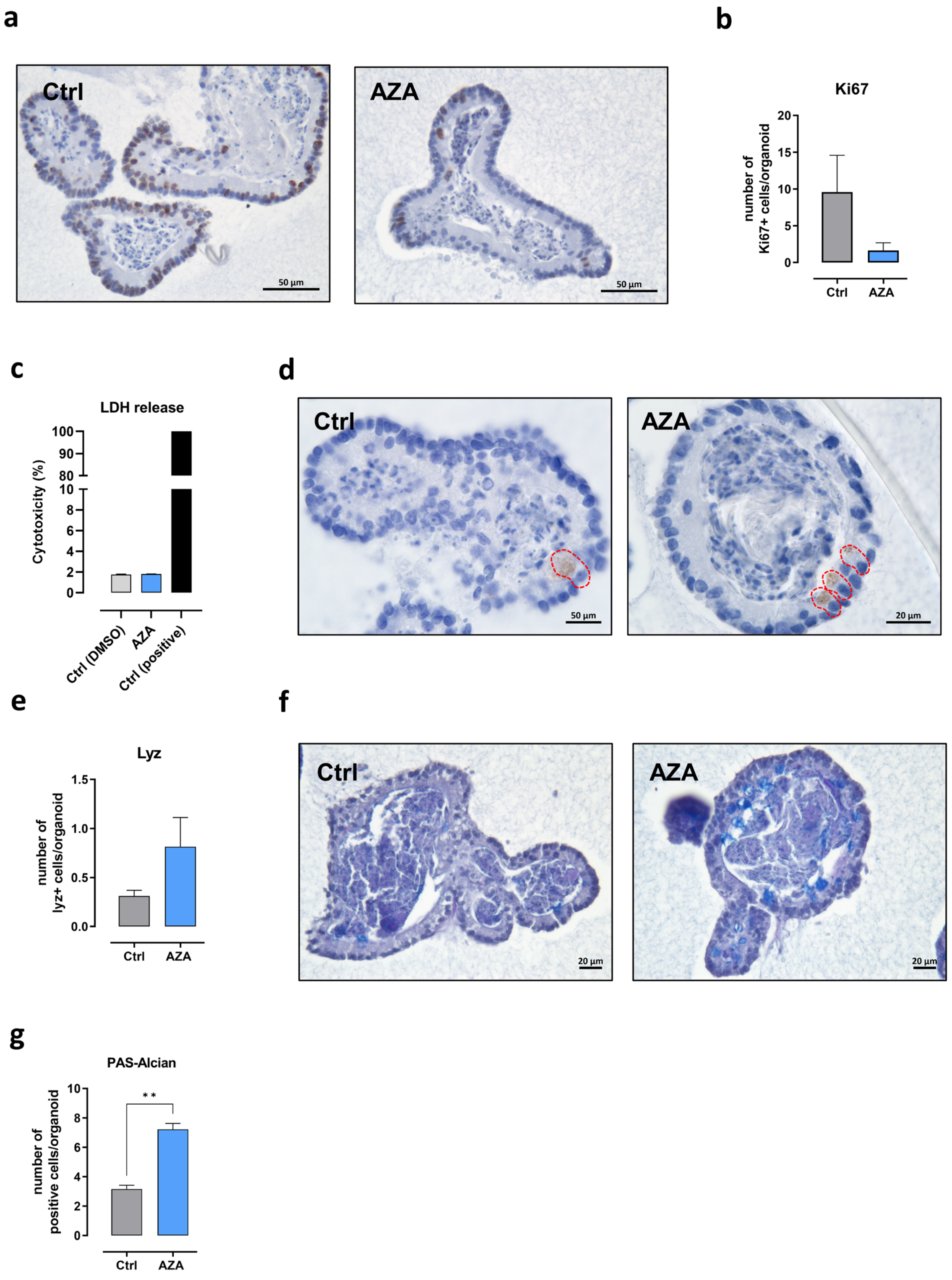
a
Ctrl
50 µm
AZA
50 µm
b
Ki67
number of Ki67+ cells/organoid
20
15
10
5
0
Ctrl
AZA
c
LDH release
Cytotoxicity (%)
100
90
80
10
8
6
4
2
0
Ctrl (DMSO)
AZA
Ctrl (positive)
d
Ctrl
50 µm
AZA
20 µm
e
Lyz
number of lyz+ cells/organoid
1.5
1.0
0.5
0.0
Ctrl
AZA
f
Ctrl
20 µm
AZA
20 µm
g
PAS-Alcian
number of positive cells/organoid
10
8
6
4
2
0
**
Ctrl
AZA

# Figure S3

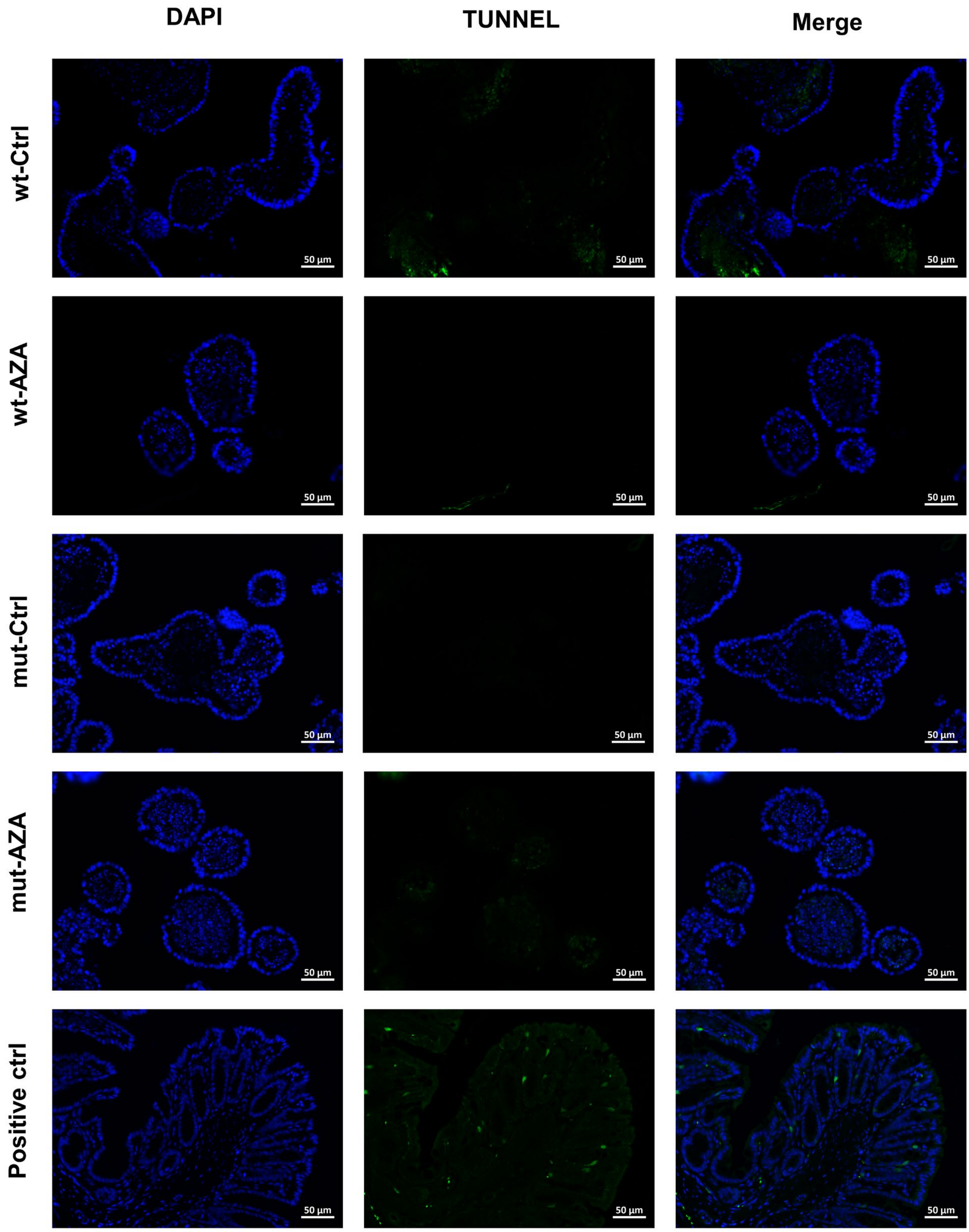
DAPI
TUNNEL
Merge
wt-Ctrl
wt-AZA
mut-Ctrl
mut-AZA
Positive ctrl
50 µm

# Figure S4

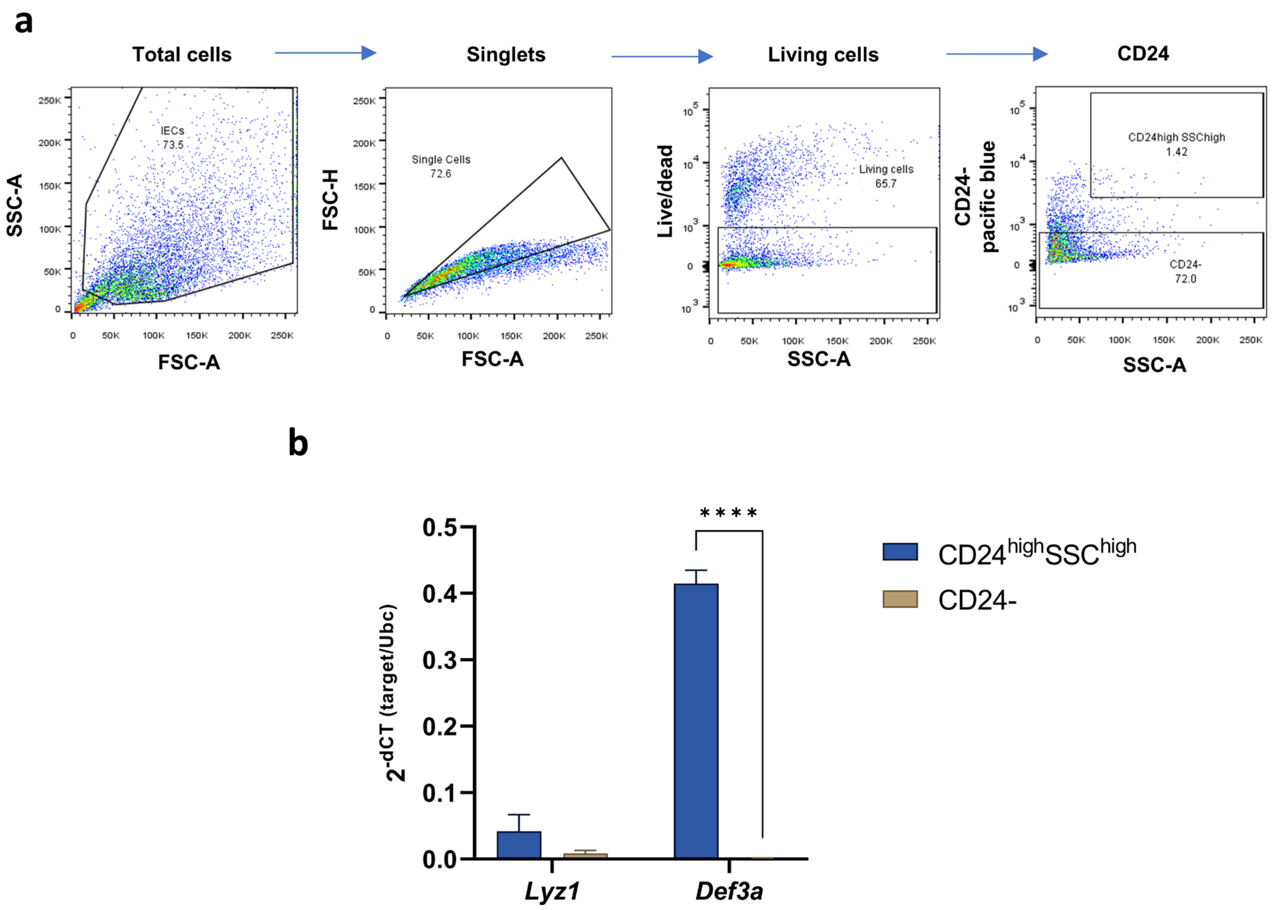
a
Total cells
Singlets
Living cells
CD24
SSC-A
FSC-A
IECs
73.5
FSC-H
Single Cells
72.6
Live/dead
SSC-A
Living cells
65.7
CD24-pacific blue
CD24high SSChigh
1.42
CD24-
72.0
b
2-dCT (target/Ubc)
****
CD24highSSChigh
CD24-
Lyz1
Def3a

# Figure S5

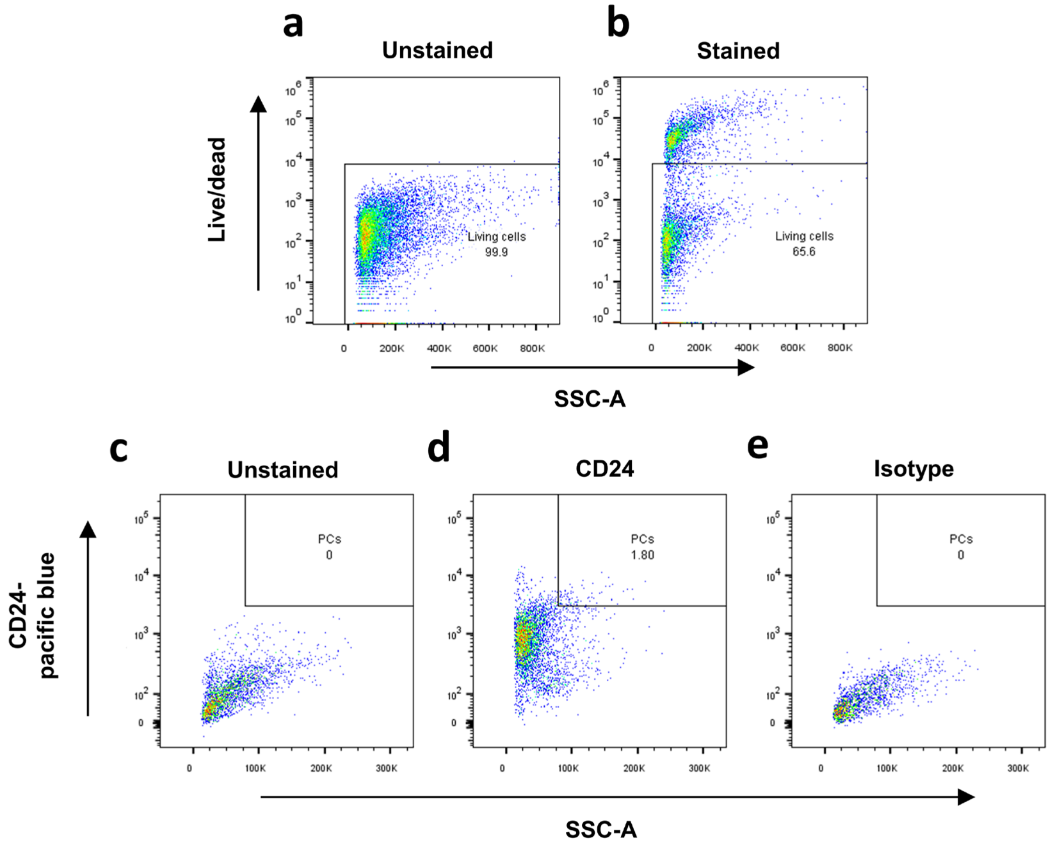

a
Unstained
b
Stained
Live/dead
Living cells
99.9
Living cells
65.6
SSC-A
c
Unstained
d
CD24
e
Isotype
CD24-
pacific blue
PCs
0
PCs
1.80
PCs
0
SSC-A
