# Supplementary figures and images for: Azathioprine promotes intestinal epithelial cell differentiation into Paneth cells and alleviates ileal Crohn’s disease severity
Source: Sci Rep. 2024 Jun 5;14:12879. doi: 10.1038/s41598-024-63730-4 (PMC11153537; doi:10.1038/s41598-024-63730-4)

**Fig. 2g**

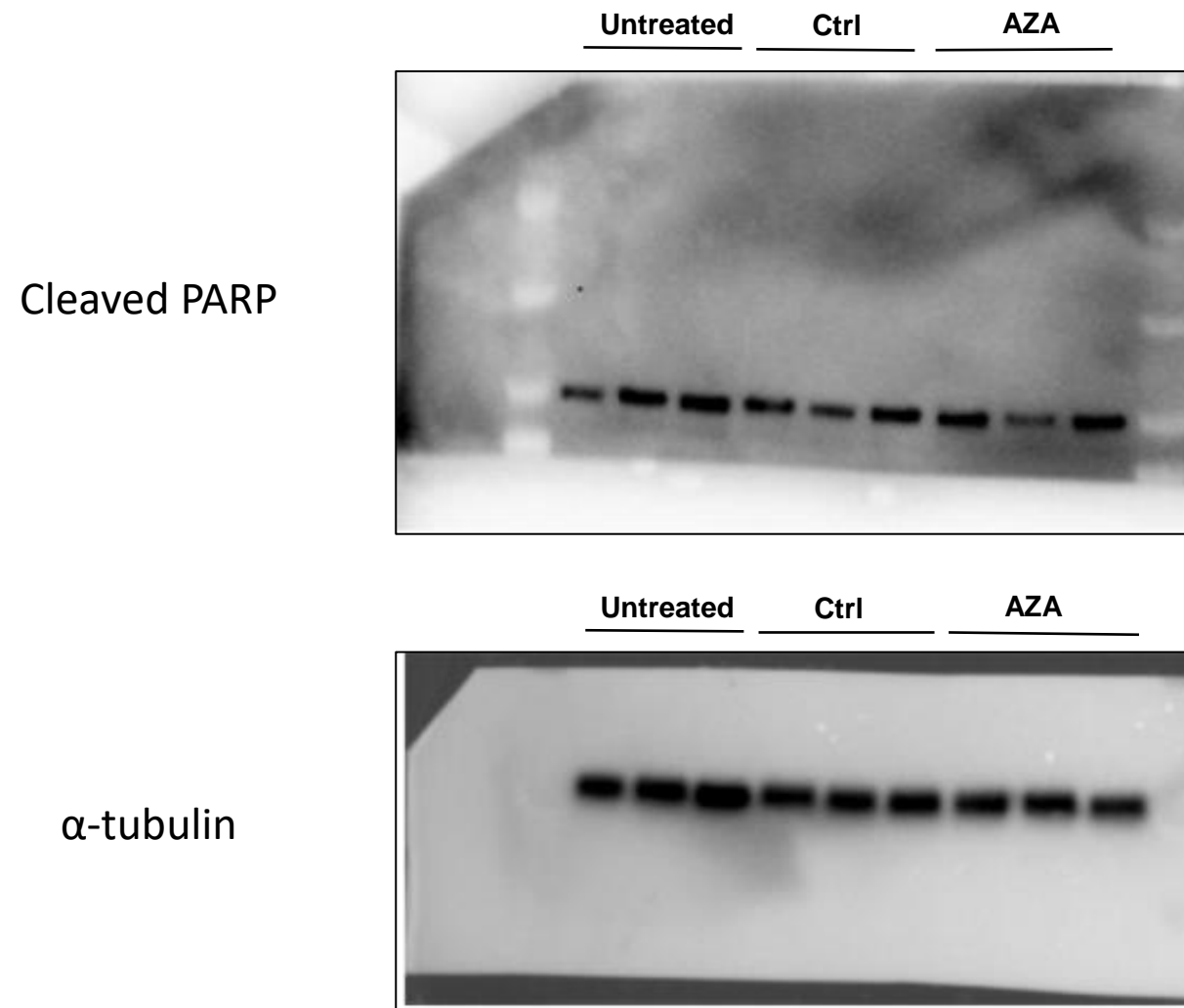

Supplement: Supplementary file 2 — Supplementary Information. [file 41598_2024_63730_MOESM2_ESM.pdf]
